# River channel change can affect flood hazard and impact substantially

**DOI:** 10.1038/s43247-026-03517-9

**Published:** 2026-05-05

**Authors:** Laurence Hawker, Stephen E. Darby, Louise Slater, Daniel R. Parsons, Richard J. Boothroyd, Philip J. Ashworth, Hannah Cloke, Pauline Delorme, Solomon H. Gebrechorkos, Helen Griffith, Yukiko Hirabayashi, Julian Leyland, Yinxue Liu, Stuart J. McLelland, Jeffrey Neal, Andrew P. Nicholas, Greg Sambrook Smith, Chris Sampson, Ellie Vahidi, Michel Wortmann, Dai Yamazaki

**Affiliations:** 1 School of Geographical Sciences, University of Bristol, Bristol, UK; 2 School of Geography and Environmental Science, University of Southampton, Southampton, UK; 3 School of Geography and the Environment, University of Oxford, Oxford, UK; 4 Geography and Environment, Loughborough University, Loughborough, UK; 5 Department of Geography and Planning, School of Environmental Sciences, University of Liverpool, Liverpool, UK; 6 Department of Geography, Faculty of Environment, Science and Economy, University of Exeter, Exeter, UK; 7 Department of Meteorology, University of Reading, Reading, UK; 8 Department of Geography and Environmental Science, University of Reading, Reading, UK; 9 Energy and Environment Institute, University of Hull, Hull, UK; 10 JBA Consulting, Skipton, UK; 11 Department of Civil Engineering, Shibaura Institute of Technology, 3-7-5 Toyosu, Koto-ku Tokyo, Japan; 12 School of Architecture, Building and Civil Engineering, Loughborough University, Loughborough, UK; 13 School of Geography, Earth and Environmental Sciences, University of Birmingham, Birmingham, UK; 14 Fathom, Clifton Heights, Bristol, UK; 15 ECMWF, Reading, UK; 16 Institute of Industrial Science, The University of Tokyo, Tokyo, Japan

**Keywords:** Natural hazards, Hydrology, Geography

## Abstract

More than one billion people are exposed to flood risk globally, with this number projected to double by 2050. Global flood models underpin risk assessment and adaptation planning, yet typically assume that river bankfull capacity corresponds to a fixed two-year return period, neglecting spatial and temporal variability in channel characteristics. Here, we evaluate how inundated areas and population exposures respond when forced with empirically-derived bankfull capacities in the Mississippi basin using the Fathom Global Flood Model. We find that present-day bankfull flows generally correspond to return periods of less than one year, leading to systematic underestimation of flood extent (9–152%) and exposure (15–472%) across 5-, 20- and 100-year flood events, with the largest discrepancies for more frequent floods. We further show that historical changes in channel morphology can influence flood impacts at magnitudes comparable to projected climate change over multi-decadal timescales, depending on emission scenarios. Our work highlights a key structural limitation in current global flood modelling frameworks with implications for risk assessments.

## Abstract (web summary)

Changes in river channel flow conveyance caused by morphodynamic processes can influence flood predictions, sometimes to a degree comparable with hydroclimatic factors, according to observational and modelling analyses.

## Introduction

Flooding has devastating impacts on people and the environment. During 2002–2021, flood events globally impacted >80 million people and caused average annual financial losses exceeding US$41 billion^1^. Moreover, flood hazard is projected to grow in the future, driven in part by the increasing frequency of extreme precipitation events under human-induced warming^2^. The number of people and value of assets exposed to flooding is also projected to increase substantially due to demographic changes, economic growth and encroachment of people into flood-prone areas^3^. For example, it has been estimated that by 2050, over two billion people will live in areas where the annual probability of flooding exceeds 1%^4^. Within this context, the accurate prediction of flood hazard and impacts is essential to enable effective policy interventions such as the development of early warning systems^5,6^ and insurance evaluations^7,8^.

**Fig. 1:**
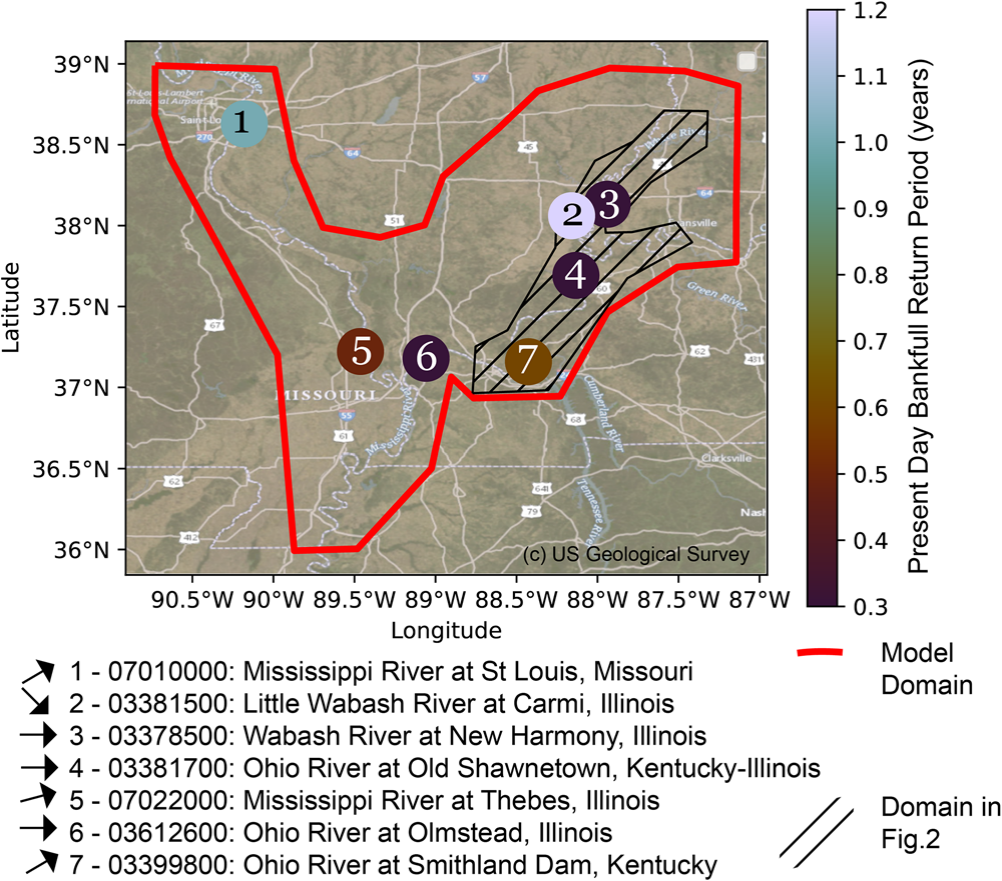
Map showing the extent of the model domain (red boundary line) used to simulate part of the 2011 Mississippi River flood event, showing locations of stream gauges employed in the study. Empirically estimated Bankfull Flow Return Periods (BFRPs; indicated by the colours of the symbols) and trends in channel conveyance capacity (indicated by directions of the arrows, with an arrow facing up showing an increase in bankfull channel capacity, horizontal no/little change and down a decrease in channel conveyance capacity) during the period of record are shown for each gauge (detailed analyses in Fig. 2 and Supplementary Fig. 1). The hashed area shows the area of the model domain highlighted in Fig. 3. Basemap data © 2025 USGS.

Flood models have substantially improved over the past decade, with enhancements in skill being driven by improved digital elevation datasets^9,10^, better estimates of regional extreme events^11^, advances in computation^12^, and more sophisticated process representation^13–15^. However, our ability to model future changes in flood hazard and risk remains poor. Local-scale modelling incorporates detailed representations of relevant hydraulic processes and provides high fidelity information, but their spatial coverage is limited, rendering their application to regional or national scale analyses challenging. Global-scale flood models (GFMs) offer a way to fill that coverage gap^16,17^ and have been employed in applications ranging from national-scale climate risk assessments^18,19^, humanitarian response planning^3,5^ and catastrophe risk evaluations^20^. However, these larger-scale models are frequently challenged by data scarcity, and a critical limitation is that existing GFMs represent river morphology in unrealistic ways. For example, GFMs are initialised by representing channel morphological properties, such as depth and roughness, by assuming that the bankfull flow-carrying capacity of the river channel can be equated to a discharge with a specified return period (RP) that is spatially and temporally^21^ invariant, and which is typically assumed to take a value of 2-years^13^. This bankfull discharge is sometimes referred to (as in this paper) as the channel conveyance capacity. As such, GFMs view river channels essentially as ‘static pipes’, in which channel conveyance varies neither through space nor time. Yet, as discussed further below, observations show that this assumption of spatial invariance is incorrect (Fig. 1) and many rivers are also undergoing morphological changes^22^ that are substantial enough to affect channel conveyance capacity and thus potentially alter flood hazard^23–25^. However, the extent to which spatial and temporal variations in channel conveyance capacity propagate through to biased GFM predictions of flood hazard and impacts remains unknown. Specifically, the direction and scale of the impacts arising cannot readily be estimated, so it is important to quantify the emergent behaviour arising from including variations in channel conveyance capacity within GFMs.

## Results and discussion

To address this question, here we quantify how: (i) different model-imposed bankfull conveyance capacities, relative to the commonly assumed 2-year RP, and (ii) multi-decadal changes in bankfull conveyance, driven by river channel evolution, affect GFM predictions of flood hazard and impacts. To do this, we employ the Fathom Global Flood Model^13,19^ (see Methods) to a 135,000 km^2^ region of the Mississippi River floodplain, where approximately 10 million people and US$3.4 billion of assets are at risk of flooding. The Fathom model is similar to other GFMs in its representation of channel conveyance (Methods), but the version of the model employed here is a regional-scale model developed for use specifically across the United States. We focus on the Mississippi floodplain as a case study because this region has experienced many floods, most notably during spring 2011^26^, an event which had an estimated return period of approximately 80 years (Supplementary Table 1). We evaluated the performance of the Fathom model simulations for the 2011 flood by comparing simulated inundation extents against those estimated from remotely sensed imagery (within the validation region delimited by the hashed area in Fig. 1) (see Methods). This comparison yielded Critical Success Index (CSI; see Methods) scores ranging between 0.584 and 0.602 depending on the imposed bankfull conveyance capacity (Supplementary Table 2). These are relatively high CSI scores (see Methods), lending confidence in the Fathom model’s ability to simulate flooding within the study area accurately. We then used the validated Fathom model in a sensitivity analysis that varied imposed bankfull return periods (BFRPs) while holding all other variables constant. This sensitivity analysis was repeated for three different driving flow discharges, chosen to represent relatively frequent (5-year return period flow), moderate (20-year return period) and much larger (100-year) floods, respectively.

**Fig. 2:**
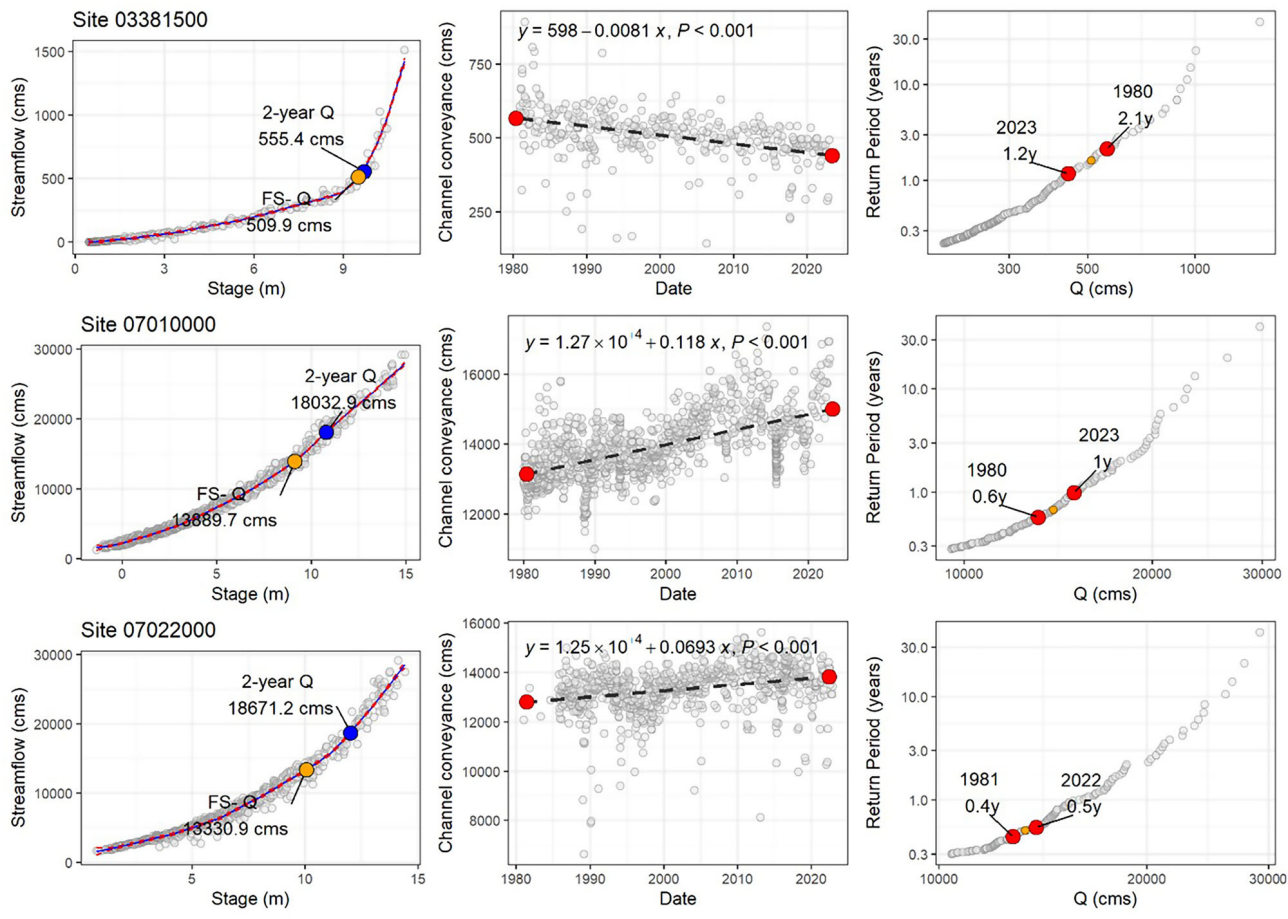
Examples of three gauges (rows) in the study region with decreasing (03381500) and increasing (07010000 and 07022000; rows) significant (*p* < 0.05) trends in bankfull channel capacity over time. *Left column* shows the transect measurements of stage and streamflow with a fitted Loess curve (blue line) and confidence intervals (red dashed lines), along with the estimated streamflow value at flood stage (“bankfull channel capacity”, orange circle) with an empirically estimated two-year exceedance flow (blue circle). *Middle column* shows channel capacity values over time, with the first and last values (red circles) extracted from the fitted linear regression (blue line). *Right column* shows estimated bankfull return periods at the start and end of the record.

The range of BFRPs (1.1–10 years) employed in our sensitivity analyses was selected to include values above and below the typically assumed value of 2-years. We also determined the actual range of empirically-derived present-day BFRPs within our study region, using United States Geological Survey (USGS) stream gauge data (Fig. 1). Specifically, for each gauge we estimated (see Methods): (i) bankfull discharge flows; (ii) their present-day return periods, and (iii) the evolution of these return periods through time. The present-day BFRP values so-obtained are *not* spatially invariant (Fig. 1), with values ranging from 0.3 to 1.2 years, a substantial deviation from the often-cited 2-year value. Four of the seven gauges within the model domain also exhibit statistically significant (*p* < 0.05) *temporal* trends in BFRP (three examples are shown in Fig. 2; see Supplementary Fig. 1 for all gauges). We return to the significance of these trends below.

**Fig. 3:**
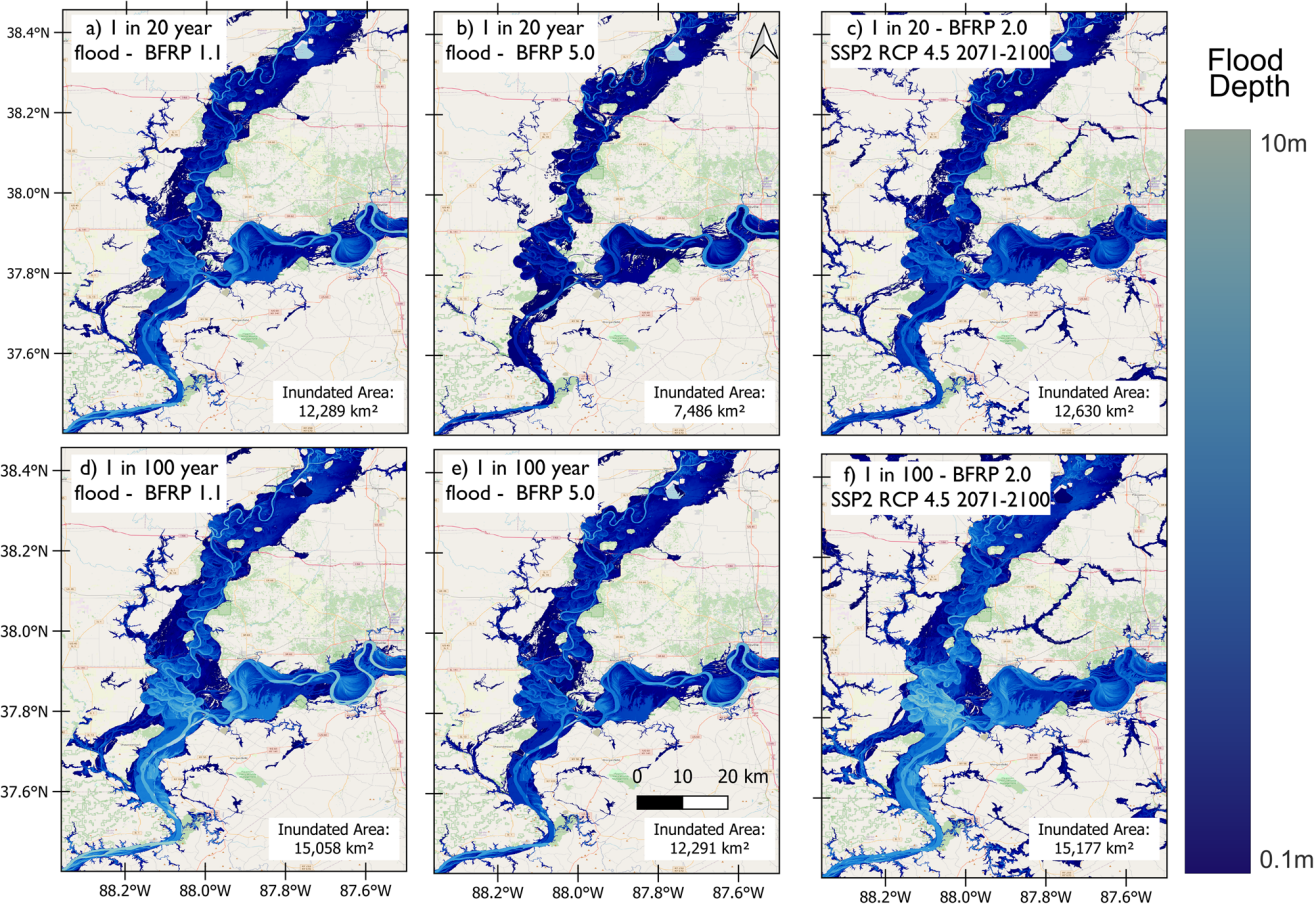
Inundation maps under varying climate forcing and channel conveyance capacities. Inundation maps under varying climate forcing and channel conveyance capacities as represented by bankfull return periods (BFRP) of 1.1 (**a**, **b**) and 5.0 (**d**, **e**) years, for discharges corresponding to the 20 (panels a-c) and 100-year (**d**–**f**) floods for the hashed area shown in Fig. 1. **c**, **f** correspond to flooding in 2071–2100 for the SSP 2 RCP 4.5 climate change scenario from ref. ^16^, and with an imposed BFRP of 2 years. Note that the inundated areas annotated on each panel refer to results for the whole model domain (the red area in Fig. 1) and not just for the zoomed-in sections shown. Dominant flow direction is north to south. Basemap data © OpenStreetMap contributors.

To provide an initial illustration of the substantial impact of the choice of model-imposed bankfull conveyance on flood hazard, Fig. 3 maps flood depths and extents simulated by the Fathom model for BFRPs of 1.1 and 5.0 years under the 20- and 100-year forcing flows. For both these flood magnitudes, the simulated inundation is greater when the smaller BFRP flow is used in the model, due to the out-of-bank flows being reached sooner (simulated flood extents are annotated on each panel of the figure). Note that the difference in flood extents simulated across the two different BFRPs plotted here is greater for the moderate (20-year) than the extreme (100-year) flood (4803 and 2767 km^2^ respectively; see Fig. 3a, b versus Fig. 3d, e). That is, the model flood inundation extent sensitivity to changes in river channel capacity is greater for smaller floods. This reflects the diminishing proportion of the total flow conveyed within the river channels, as opposed to over the floodplain, as flood magnitude increases. For reference, Fig. 3 also highlights the flood inundation extents simulated for a future (2071–2100) climate change scenario (Shared Socio-economic Pathway 2/Representative Concentration Pathway 4.5; see Methods) and using an imposed bankfull return period of 2-years. We use these climate change projections later to compare their impacts in driving Fathom GFM simulated flood extents to those forced by changing channel conveyance.

**Fig. 4:**
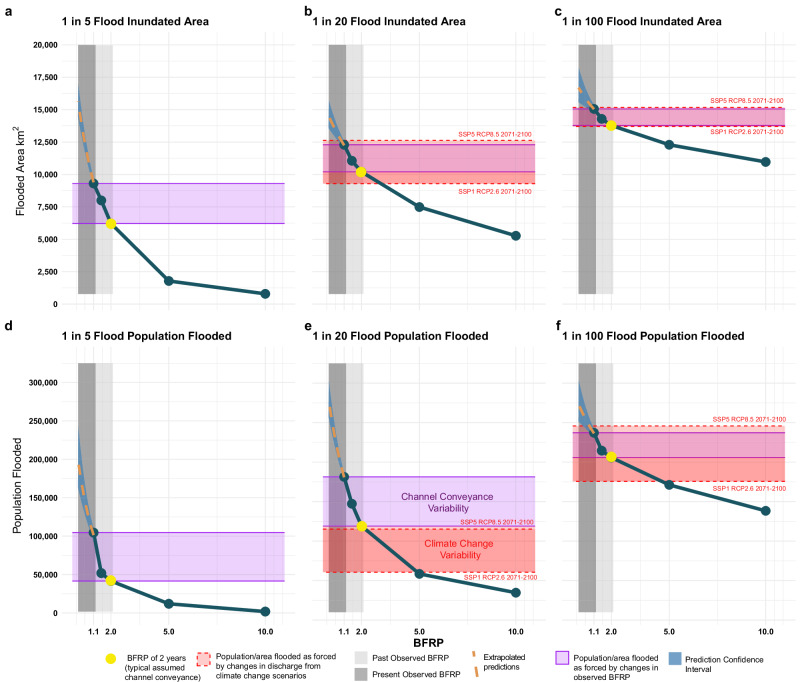
Sensitivity of inundated area and population exposed to different bankfull return periods for three different return period floods. Sensitivity of simulated inundated area (top row) and population exposed to flooding (bottom row) to changes in channel conveyance capacity for 5-year (left column; **a**, **d**), 20-year (middle column; **b**, **e**) and 100-year (right column; **c**, **f**) floods (blue lines). Note that the dashed orange lines for bankfull return periods (BFRPs) less than 1.1 years are based on curve-fits to the modelled data, with the blue shaded area corresponding to computed confidence intervals (see Methods). The dark grey vertical band represents the range of present-day bankfull conveyance capacities (BFRPs) estimated at the seven stream gauges in the study area (0.3–1.2-years; see right hand column of Supplementary Fig. 1) and is compared to the 2-year bankfull return period flow (shown as a yellow symbol). The range of values within the red shading indicates simulated inundated areas/populations exposed under moderate (SSP1 RCP2.6; lower bound) and intense (SSP5 RCP8.5; upper bounds) future (2071–2100) climate change scenarios and for a bankfull channel conveyance capacity of 2-years (note that these climate change scenarios are only available for 20-year and 100-year floods). Purple shaded areas indicate simulated inundated areas/populations exposed when bankfull channel conveyance is varied according to the historical trends in BFRP observed at the stream gauges in the study area, as indicated here by the light grey vertical bands.

Figure 4 presents the full results of our sensitivity analysis, which we use initially to demonstrate the large potential model biases arising from the choice of BFRP used to represent the river channel morphology. This sensitivity analysis shows how the Fathom flood predictions vary in response to channel conveyance capacity, as indexed here using the BFRP flow. Note that we also estimate flood impact in terms of the populations exposed to flooding, as determined using WorldPop data^27^, in the simulated inundated areas (see Methods). On each diagram, the darker grey vertical bands highlight the range of present-day conveyance capacities as estimated at the stream gauges in our area of interest (see Fig. 2 and Supplementary Fig. 1). This range of empirically-derived bankfull return periods of 0.3 to 1.1 years differs substantially from the 2-year value commonly employed in GFMs. These differences, when combined with the sensitive response of the Fathom model to changes in the imposed BFRP, allow the biases in flood hazard and impact estimates arising from the choice of BFRP to be determined. Since the model sensitivity is dependent on the magnitude of the forcing flood (Fig. 3; discussed above), these computed biases are also flood-magnitude dependent, being greater for smaller floods. For example, the inundated areas simulated for the 2-year BFRP, denoted by the yellow symbols on Fig. 4, are 6220 km^2^, 10,204 km^2^ and 13,781 km^2^ for the 5-year, 20-year and 100-year floods, respectively. In contrast, when imposing the more-realistic range of observed present-day BFRP values of 1.1 to 0.3-years, simulated inundation extents increase to between 9306 and 15,654 km^2^ (a 50–151% increase compared to the 2-year BFRP), 12,289 and 14,506 km^2^ (a 20–42% increase compared to the 2-year BFRP) and to between 15,058 and 16,697 km^2^ (a 9% to 21% increase) for the 5-year, 20-year and 100-year floods, respectively (Fig. 4a–c and Supplementary Table 3). In terms of flood impacts, the computed biases are even greater. Specifically, for the 20-year flood the number of people exposed increases from 119,059 for the baseline 2-year BFRP scenario to between 181,283 and 278,244 (a 52–133.7% increase across the range of present-day observed BFRPs), while for the 100-year flood the number of people exposed grows from 205,817 for the baseline 2-year BFRP scenario to between 237,162 and 273,296 (a 15 to 33% increase) (Fig. 4e, f and Supplementary Table 3). For the more frequently recurring 5-year flood, the computed biases of people exposed as a percentage are even greater, at between 104,588 and 197,865 (a 149 to 472% increase). Note that the upper end of the bias ranges quoted here, which corresponds to the 0.3-year BFRP, is uncertain. This is because, like other large-scale flood models, the Fathom model used here is unable to represent bankfull return periods below 1.1 years (see Methods), so our simulations are thus truncated at this threshold. We therefore estimated the flood inundation extent and populations exposed for BFRPs below 1.1 years via extrapolation (dashed lines, with confidence intervals shaded in blue on Fig. 4) along best-fit curves to the model results (see Methods).

Finally, we undertake an indicative comparison of the relative effects of *temporal* changes in river channel conveyance capacity versus future climate change on flood inundation and population exposure. To do this, we first quantified the influence of future climate by adjusting the return periods of the two (20- and 100-year) present-day flows driving the Fathom model for the period 2071–2100^16^, based on the Coupled Model Intercomparison Project Phase 6 (CMIP6) climate simulations (see Methods). For our study area, our three future climate scenarios show moderate variations in return period, with the SSP1 RCP2.6 scenario showing a decrease in return period (Supplementary Figs. 2 and 3). The change in inundated area and people exposed between the present day and future (2071–2100) climates, when imposing a 2-year RP channel conveyance capacity, is then indicated by the red shaded areas on Fig. 4 (with numeric values listed in Supplementary Table 3). To quantify the possible impacts of river channel evolution on flood extent and exposed populations, we imposed BFRPs of 2.1 and 1.2-years within the model. While we do not claim that this imposed reduction in channel conveyance is representative of the actual historical evolution of the river channel network in our model domain over the last four decades, we argue that the magnitude of the imposed change is plausible, and perhaps even conservative. This is because the selected range of imposed BFRPs is consistent with historical data between the years 1980 and 2023 as observed at the stream gauges in our study area (see top right panel of Fig. 2). Yet, stream gauges are often sited at locations believed to be relatively stable and so morphological changes elsewhere could even be larger. Nevertheless, we frame this part of our analysis as an indicative comparison only.

The past changes in channel conveyance, represented by the light grey shading on Fig. 4, force changes in inundated area and population exposure as indicated by the purple shading on the diagrams (with numeric values listed in Supplementary Tables 4, 5 and 6 for the 5-, 20- and 100-year forcing flows, respectively). A comparison of the red versus purple shaded areas thus indicates the potential relative effects of ~6 decades of potential future climate change versus ~4 decades of past channel evolution on inundation extent and populations exposed. For the moderate (20-year RP) driving flow discharge, the declining bankfull capacity results in a substantial predicted historical increase in flood extent of 403 km^2^ per decade that is comparable, or greater, in magnitude to the changes forced by future climate change, which range from −150 km^2^ (SSP1 RCP2.6) to +404 km^2^ per decade (SSP5 RCP8.5) across the forcing climate scenarios (Supplementary Table 5). Note that the increases in flood extent due to the imposed reductions in channel capacity potentially offset the declines in flood extent simulated for the two more moderate future climate scenarios, but compound the increase projected for the higher intensity (SSP5 RCP8.5) climate change scenario. This pattern of response is evident also for the more extreme (100-year RP) driving flow discharge, albeit with more muted rates of change: +262 km^2^ per decade under the morphological forcing and −13 to +233 km^2^ per decade (depending on scenario) under future climate change (Supplementary Table 6). Changes in flood impact forced by past morphological versus future climate changes are also similar in magnitude. For example, for the moderate (20-year RP), the number of people exposed to flooding increases by 12,523 per decade due to the imposed reductions in bankfull capacity, but declines between 9,741 and 641 per decade (depending on scenario) under future climate change (Supplementary Table 5). For the more extreme (100-year RP) flood, the increase in flood impact due to the imposed channel evolution ( + 6,077 people per decade) is likewise comparable to the change (−5,037 to +6,664 people per decade, depending on scenario) forced by future climate change (Supplementary Table 6). Inundation extent and population exposed were also calculated for the relatively frequent flood (5-year RP) (Supplementary Table 4), but could not be compared to climate change simulations as the climate forcing was not available for this return period. Other non-climatic drivers that can markedly influence channel conveyance and flood hazard, such as land-use change, sedimentation, urbanisation and dam operations, were not possible to model and isolate in the context of this study, but are still important and should be explored further in future work.

Overall, our study shows that channel conveyance capacity variability matters in estimating flood hazard and impacts. We find that neither of the common assumptions that GFMs employed to represent channel conveyance capacity (using a bankfull discharge with a return period that is spatially invariant and which has an assumed value of 2 years) is supported by empirical evidence. By running our Fathom model simulations across the range of present-day bankfull return periods actually observed in our study area, we obtain substantial bias in simulated flood hazard, with inundated areas estimated to be between 50 and 152% greater for the relatively frequent 5-year flood, between 20 and 42% greater for a moderate 20-year flood, and between 9 and 21% greater for a more extreme 100-year flood, when using more realistic bankfull return periods informed by gauge data. Biases in flood impacts are even greater, with the number of people estimated to be exposed to flooding between: (i) 149 to 472% greater for a relatively frequent 5-year flood; (ii) 52 and 118% greater for a moderate 20-year flood, and (iii) 15 and 54% greater for the 100-year flood. An ancillary finding is that the effect of historical evolution of channel morphology on flood hazard and impacts is potentially greater in our case study area than the effect of future climate-driven changes under a low (SSP1 RCP2.6) and a moderate (SSP2 RCP4.6) emissions scenario, for both moderate (20-yr RP) and extreme (100-yr RP) floods. Even under a high emissions scenario (SSP5 RCP8.5), the evolution of flood hazard and impacts forced by future climate change is approximately equal in magnitude to the evolution of flood extent and exposure forced by imposed changes in bankfull capacity that are comparable to those observed in the study region in the last four decades.

While we acknowledge that our study focuses on a single study area and employs a specific, regional-scale variant of a GFM, we argue that the implications of our findings are broadly applicable. This is because the Fathom model estimates channel conveyance in a similar way to other GFMs^14,28,29^, implying that their model sensitivities are likely similar to those quantified here. Moreover, recent work has shown that many other rivers, not just those in the Mississippi region, have bankfull flow return periods that deviate substantially from 2 years. Specifically, Liu et al.^30^ have shown that the majority (64.6%) of 8519 gauging station locations worldwide have bankfull flows with return periods that are either less than 1-year or greater than 3-years. Moreover, other work (Slater et al., 2015)^23^ has shown that temporal changes in bankfull return periods occur across multiple stream gauge locations in the United States, not just at the specific locations employed in our study. This means that the existing approach of GFMs utilising a temporally and spatially invariant 2-year flow return period to represent bankfull conveyance capacity is incorrect and likely to lead to substantial biases in estimates of flood hazard across many of the world’s floodplains. As such, we now need to employ emergent datasets such as Liu et al.^30^ to provide new and improved representations of the conveyance capacity of river channels, and incorporate these with new river networks that map bifurcating flows^31^. In addition, water-surface profiles from the Surface Water and Ocean Topography (SWOT) satellite^32,33^ offer great potential to enhance model calibration and bathymetry inversion. However, these opportunities essentially offer a better way to characterise a static snapshot of the river morphology, and therefore, there is an urgent need to generate time-varying estimates of conveyance capacity globally within GFMs in order to complement these advances and expand the scope of the ‘proof of concept’ investigation that we present here to the global river network.

## Methods

### Flood model simulations

Flood inundation was simulated using the Fathom Global Flood Model (GFM)^13,19^ at 1 arc second ( ~ 30 m) grid spacing. The model is underpinned by LISFLOOD-FP, which solves the local inertial formulation of the shallow water equations^14,34^. The version of the model developed in this study is the same as ref. ^19^. Elevation data is predominantly taken from the USGS National Elevation Dataset (NED) at 1 arc second grid spacing ( ~ 30 m), augmented with LiDAR terrain data where available. River channels are modelled in one dimension, which allows for river channels of any size to be represented (even below model grid spacing). The location of rivers is delineated by a flow accumulation grid created from the elevation data and USGS National Hydrography Dataset. Even in this relatively well-measured region, river bathymetry is largely unknown, and we thus parametrise channel conveyance by assuming the channel can convey a certain return period discharge. Normally, the channel conveyance is assumed to be the 1 in 2-year return period flow, but herein we undertake a sensitivity analysis in which we alter the channel capacity to convey 5 different return period flows: 1.1, 1.5, 2, 5 and 10 years, respectively. Bed elevation is then estimated using an inverted gradually varying solver^35^ with bankfull elevation extracted using a 3 × 3 moving window from land pixels from the DEM adjacent to the water mask. Channel roughness is fixed at a Manning’s coefficient of 0.035, and bathymetry is estimated given this assumption, making the model less sensitive to Manning’s n than a typical flood model based on observed geometry. Floodplain roughness is fixed based on land cover as outlined by Sampson et al.^13^, these can vary in space but are fixed for all simulations. Lastly, return period discharges, used for both channel bed estimation and flow input for flood simulations, were determined using a regional flood frequency analysis (RFFA). We used the methods and dataset of Bates et al.^36^, which took the annual maxima discharge from 6,902 USGS river gauges and grouped them by proximity and hydrologically similarity to calculate index flow for every pixel in the flow accumulation array. Note that, by using the annual maxima discharge, we could not calculate flow return periods below 1 year. Consequently, the bankfull channel capacity used in the flood model could not match the values estimated empirically from the gauges (see *Estimation of Bankfull Return Periods*, below). This is an inherent limitation of the RFFA used to drive the model. Using the peaks-over-threshold (POT) approach to calculate extreme values could theoretically be used to calculate bankfull return periods below 1.1 years and thus negate the need to extrapolate results below 1.1 years, but it was out of the scope of this study to re-calculate bankfull return periods using this method, especially as the annual maxima approach is widely used in GFMs. Extreme flows are estimated by deriving growth curves from the pooled gauges, with a 6% and 29% error for the 10-year and 100-year flows, respectively^36^. However, this error is dampened by coupling the channel conveyance estimate and RFFA, as the channel conveyance will account for an over- or underestimate of extreme flow by the RFFA (i.e., if the RFFA is underestimated, then the channel conveyance will be lower). Return-period discharges are derived from RFFA for which only basin-scale performance metrics are available; gauge-specific uncertainties cannot therefore, be quantified. While systematic bias in these estimates may affect absolute flood magnitudes, the analysis focuses on relative changes in flood hazard associated with channel-depth change, which are far less sensitive to absolute return period flow values, such that explicitly propagating return flow uncertainty would not alter the direction or relative magnitude of the reported effects. We simulated 3 return period flows: the 1 in 5, 1 in 20 and 1 in 100-year return period flows for the 5 channel conveyance capacities (i.e., channels able to convey the 1 in 1.1; 1.5; 2; 5; and 10-year flows). These three driving return period flows were chosen as they represent a relatively frequent flood (1 in 5-years), a moderate flood (1 in 20-years) and the flood defence return period flow (1 in 100 years).

### Model validation

To test the quality of the model, we compared simulations of the 2011 flood event to inundation extents observed by remote sensing. Google Earth Engine was used to estimate flood inundation extents from Landsat 5 Thematic Mapper imagery acquired during the 2011 flood. Partially cloud-free imagery was available for sections of the Mississippi River at Wabash-Ohio on 3rd May 2011 and Cairo-Tiptonville on 10th May 2011. The CFmask algorithm was applied to mask obstructions from cloud and cloud shadow pixels^37^, and the modified normalised difference water index (MNDWI^38^) was used to indicate the presence of water. Based on manual testing, a MNDWI threshold of 0 was defined to discriminate between water and non-water pixels^39^. To ensure that water pixels were associated with floodwaters from the main channels, only water pixels connected to centerlines from the Global River Widths from Landsat (GRWL) Database^40^ were included in the Landsat-derived flood footprints used for model validation purposes.

The model simulations used in the validation were driven by a flow discharge corresponding to the 1 in 75-year return period flow to match, as closely as possible, the estimated return period of the 2011 event. The Fathom model operates on return-period flood rather than event-specific hydrographs, and thus we compared to the closest return period of the Fathom model. In fact, calculated Annual Exceedance Probabilities (AEPs) exceed 0.2 within some of the tributary channels, but with an average of 2 to 4 within the Mississippi mainstem (see Supplementary Table 1). Nevertheless, an estimated AEP within the test domain, based on data from 5 gauges, results in an estimated mean AEP of ~1.2 (95% Confidence Intervals of 0.75 and 3.85), and thus a representative flood reoccurrence interval of ~80 years for the event^41^ (Supplementary Table 1). The 1 in 75-year flood return period was used in the simulation as this return period is the closest available within the suite of existing GFM simulations employed in this study^13^. We acknowledge that we are using a spatially varying return period event to compare against a spatially invariant return model simulation; the skill scores should only be viewed as indicative of simulation quality. Within the validation itself, we employed the widely used Critical Success Index (CSI) metric^42^ to assess the skill of the simulations (see Supplementary Table 2), as the CSI accounts for both under and over-prediction, with a score of 0 indicating no skill and a score of 1 indicating a perfect model. However, CSI scores above 0.7 are rarely obtained^43^. For regional scale models like the one used in this study, CSI scores of 0.56–0.67^13^, 0.78^36^ and 0.65–0.76^18^ have been obtained in prior studies. Unlike local-scale models, these regional-scale (and indeed global-scale models) have not been extensively calibrated to local conditions. Other metrics reported in Supplementary Table 2 were computed following Wing et al. (2017)^44^. These metrics include the Hit Rate (HR, ranges from 0 indicating no skill to 1 indicating good skill), which tests the proportion of the observed wet areas replicated by the model and thus measures the model’s tendency to underpredict flood hazard; the Miss Rate (defined as 1 – HR), and; the False Alarm Ratio (FAR, ranges from 0 indicating no false alarms to 1 indicating all false alarms), which indicates the proportion of the observed wet areas that are not inundated in the model and thus measures the model’s tendency to overpredict flood extent.

### Estimation of bankfull return periods and their change through time

Streamflow transect data were downloaded for all gauges within the model domain from the US Geological Survey National Water Information System (NWIS), and bankfull stage values were obtained for each of the sites from the National Water Prediction Service of the National Oceanic and Atmospheric Administration (NWS, NOAA). Of the available gauges, one (03322420; the Ohio River at Uniontown Dam, Kentucky) was discarded from the analysis due to the lack of recent data, leaving a total of seven gauges employed in the analysis. Of these seven sites, one gauge (03381500; the Little Wabash River at Carmi, Illinois) did not have a NWS estimate of flood stage, so a value of 9.5 m was employed based on visual breakpoint analysis of the stage-discharge and stage-width transect data at that site. For all seven sites, the stage-discharge and stage-width relationships were checked visually to ensure that the flood stage corresponded to a breakpoint, with discharge or width increasing visibly above the flood stage value. However, the presence of a breakpoint in the data was more visible at some sites than others, and it is possible in some sites that the NWS moderate flood stage value is a closer approximation of bankfull. All data were trimmed to the period 1980-01-01 to present, and a small handful of extreme, clearly non-physical measurements were removed (e.g., isolated stage or width values lying far outside the observed stage-discharge relationship), as these were visually obvious artefacts and did not represent plausible channel geometry.

Channel capacity analyses were then undertaken following the methods of Slater et al. (2015),^23^ summarised here briefly. First, a locally weighted scatterplot smoothing curve (Loess) was fit to the stage-discharge transect data and used to extract bankfull discharge at flood stage based on the NWS flood stage value (see Fig. 2, left column). Next, channel capacity at flood stage was estimated at each point in time (see Fig. 2, middle column) by adding the residuals of the stage-discharge relationship to the estimated bankfull discharge. This approach assumes the channel conveyance capacity is homoscedastic and behaves similarly (e.g., increasing) across different values of stage. Channel capacity values were then plotted over time, and a linear regression was fitted to assess which sites exhibit significant trends in channel capacity. Finally, channel conveyance capacity values were extracted for the first and last years of the data from the linear regression and assumed to represent average channel capacity at the start and end dates (e.g., in 1980 and 2022) of the analysis.

Flow return periods were computed by downloading the daily streamflow data from the USGS NWIS for the period 1980-01-01 to present. To compute flow return periods, we retained only years with at least 95% complete data (i.e. more than 346 values). We used a common threshold of the 80th percentile of all daily data at each site as the baseline threshold from which to extract flood peaks (we note that the final estimated return periods are not sensitive to the choice of threshold; results using the 70th percentile differed by only 3%). Flood peaks were then extracted following the approach recommended by the Water Resource Council of the USA, as described in Lang et al. (1999)^45^, where the flood threshold separation is computed as 4 days + the logarithm of the drainage area of each site in square kilometres, with a minimal intermediate flow of less than 75% of the lowest of the two peaks. Return periods were computed by ranking the flood peaks in descending order with the ‘average’ ties method and computing (*n* + 1)/rank, where n represents the total number of identified flood peaks. A Loess curve was employed to fit the empirical relationship between the peak flow values and the estimated return periods. Finally, the return periods were extracted for the bankfull channel capacity values in the start year and end year from this empirical relationship. Note that this approach estimates the influence of changing the channel capacity relative to a stationary flow distribution; i.e., the same flow record is used for the full time period to isolate the effect of changing channel capacity^23^.

### Estimation of population exposure

We estimated the number of people living (based on 100 m spatial resolution building constrained WorldPop data for the year 2020^27^) within the flood extents simulated by the various conveyance and driving flow return period combinations. WorldPop data was downscaled to 1 arc second (to match the flood model resolution) and overlayed with binary flood maps. To create the binary flood maps, we used a threshold flood depth of 15 cm, and thus, water depth above 15 cm was considered flooded. To calculate population exposure, we used a raster calculator to multiple the downscaled population by the binary flood maps. This workflow was implemented in R. Temporal population change is not incorporated in our exposure analysis as modelling population dynamics is beyond the scope of this work, but can be an important driver of future flood exposure^46^.

### Extrapolation of flood predictors

The lowest possible bankfull return period (BFRP) used to represent bankfull channel capacity in the Fathom GFM is 1.1 years, but empirically-derived estimates from stream gauges in our study region give values as low as 0.3 years. To include these low BFRP values in our analysis, we modelled the relationship between the simulated BFRP values available in the Fathom model (1.1, 1.5, 2, 5 and 10) and the corresponding simulated inundated areas and population exposures. Initial curve fitting using standard non-linear regression approaches and information-theoretic model selection (based on Akaike Information Criterion and residual standard error) using the ‘aomisc’ and ‘drc’ packages in R, revealed that for inundated area, the Weibull 3 parameter function was the best fit and for population exposed, the asymptotic and power curve parameters were the best fit. However, these well-performing functions produced some instances of implausible extrapolations when extended below the minimum modelled BFRP (0.3), including non-monotonic behaviour and exposure estimates that were inconsistent across return periods (e.g. higher exposure for the 1-in-20-year flood than for the 1-in-100-year flood). Additionally, when estimating confidence intervals, these estimates extended into implausible negative values for inundated area and population exposed.

To address these limitations, we adopted a Bayesian non-linear modelling framework with explicit structural and distributional constraints. For both inundated area and population exposure, we fitted monotonic asymptotic models using a Gamma likelihood to enforce strictly positive predictions and to better represent heteroscedasticity in the simulated outputs. Models were anchored at the lowest available modelled BFRP (1.1 years) using a high-weight pseudo-observation to ensure that extrapolations to lower BFRP values remained consistent with the simulated data. Weakly informative, data-driven priors were specified to stabilise inference given the limited number of observations available for each return period. This approach ensured monotonic, physically plausible behaviour across the full range of BFRP values, while providing coherent uncertainty estimates for extrapolated inundation and exposure metrics. All Bayesian models were fitted using the ‘brms’ package in R^47^. Despite our best efforts and advanced approached, our extrapolations should be treated with caution as we are extrapolating using only 5 BFRP data points.

### Future climate change projections

We quantified the potential influence of typical future climate change projections on our model simulations by using the change in return period estimates from Hirabayashi et al. (2021)^16^. These altered return period estimates for the period 2071–2100 are based on the Coupled Model Intercomparison Project Phase 6 (CMIP6) climate simulations. Hirabayashi et al. (2021)^16^ estimated return periods for the past (1971–2000) and future (2071–2100) by extracting daily runoff data from multiple atmosphere–ocean general circulation models (AOGCM) from CMIP6, routing the runoff through a global river network using CaMa-Flood v4.0^48^ and fitting a two parameter Gumbel extreme value distribution. Three climate scenarios were used in the Hirabayashi et al. (2021)^16^ study (SSP1-RCP2.6, SSP2-RCP4.5, SSP5-RCP8.5) and were subsequently used in this analysis. Flood inundation projections for the end of the century (2071–2100) were then created by estimating the multi-model median return period for discharge values corresponding to both the 1 in 20 and 1 in 100-year floods over the historical period (1971–2000). These inundation maps for the end of the century (2071–2100) were created by extracting the closest return period Fathom flood map for each cell in the Hirabayashi et al. (2021) data, and stitching these maps together to form a climate change-impacted flood map that has spatially distributed changes in return periods. It should be noted that the historical period of Hirabayashi et al. (2021) (1971–2000) is different to our geomorphic analysis (1980–2023), and thus the absolute changes in flood impacts that we report should be viewed as indicative rather than absolute results. By using the multi-model median return period for discharge values from the nine AOGCMs used in the Hirabayashi et al. study, we reduce the influence of outlier AOGCM model results and minimise structure uncertainty. To estimate the return period, Hirabayashi et al. ^16^ used a Gumbel distribution and reported good accuracy in our region with a probability plot correlation coefficient (PPCC) score of >0.95 (95% confidence interval). Exploring the full range of uncertainty from AOGCM and its propagation to flood inundation was outside the scope of this indicative level study.

## Supplementary information

Transparent Peer Review file

Supplementary Material

## Data Availability

Fathom global flood model data are available for academic purposes and were provided by Fathom. The WorldPop constrained high-resolution population counts are available to download online (https://hub.worldpop.org/doi/10.5258/SOTON/WP00685). Google Earth Engine (GEE) codes to extract flood footprints from Landsat imagery for model validation are available here (https://code.earthengine.google.com/30e8f4a66cf99b815c135b87d1429946 and https://code.earthengine.google.com/09ca689dfb8e5df65f48cad5121b43c4). Gauging station data was obtained from the USGS National Water Information System (NWIS; https://waterdata.usgs.gov/nwis), with the channel capacity data retrieved using the dataretrieval R package^49^.

## Code availability

The code to extract flood footprints using Google Earth Engine (GEE) are available from (https://code.earthengine.google.com/30e8f4a66cf99b815c135b87d1429946. The custom R code to analyse the gauge data and process the flood model outputs are available upon reasonable request.
