# Supplementary material for: River channel change can affect flood hazard and impact substantially
Source: Commun Earth Environ. 2026 May 5;7(1):585. doi: 10.1038/s43247-026-03517-9 (PMC13349851; doi:10.1038/s43247-026-03517-9)
Supplement: Supplementary file 2 — Supplementary Material [file 43247_2026_3517_MOESM2_ESM.pdf]

Supplementary Information for:
River channel change can affect flood hazard and impact substantially

Laurence Hawker, Stephen E. Darby, Louise Slater, Daniel R. Parsons, Richard J. Boothroyd, Philip J. Ashworth, Hannah Cloke, Pauline Delorme, Solomon H. Gebrechorkos, Helen Griffith, Yukiko Hirabayashi, Julian Leyland, Yinxue Liu, Stuart J. McLelland, Jeffrey Neal, Andrew P. Nicholas, Greg Sambrook Smith, Chris Sampson, Ellie Vahidi, Michel Wortmann, and Dai Yamazaki

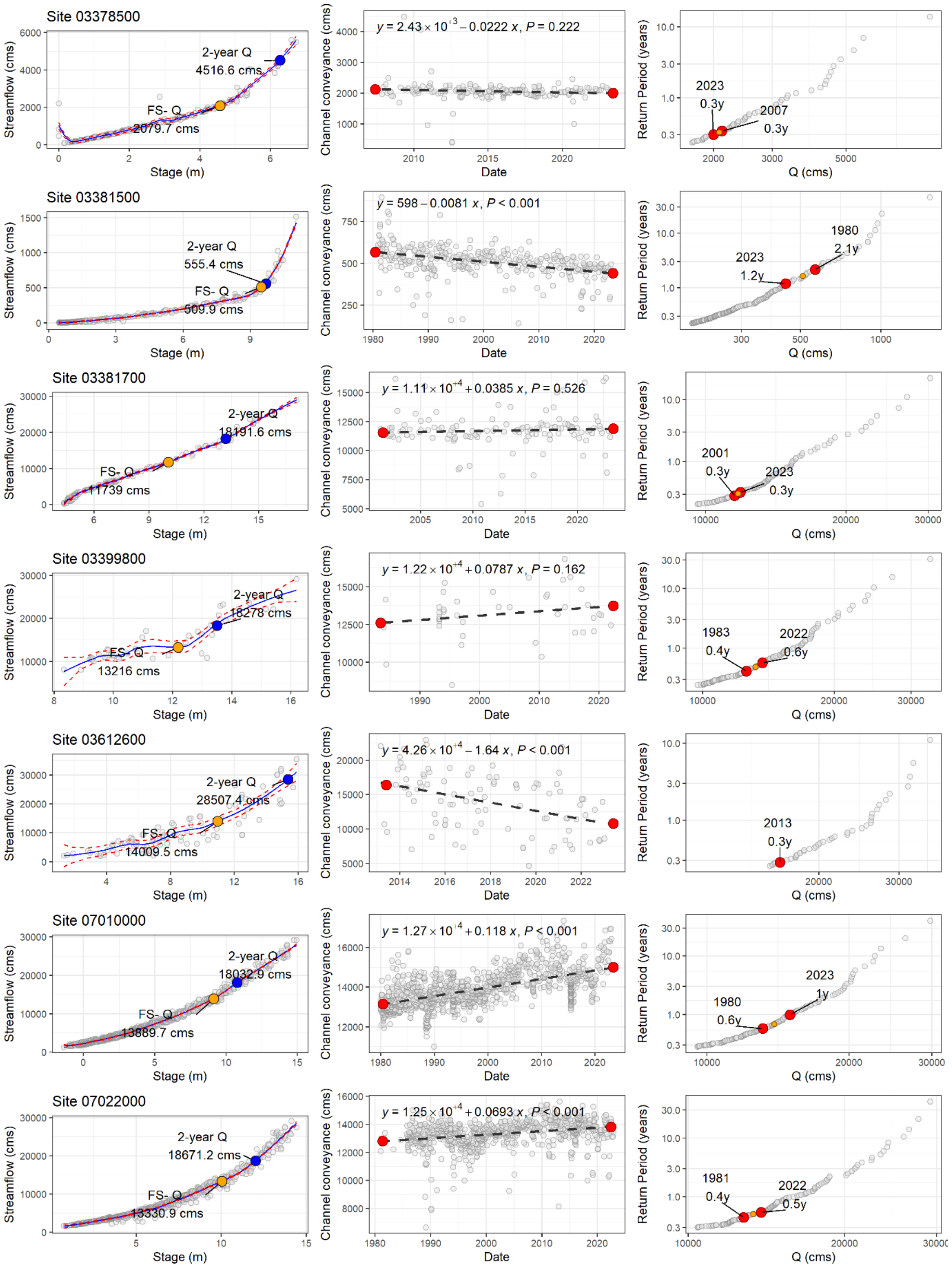

Supplementary Figure 1: *Influence of changes in bankfull channel conveyance capacity on bankfull flow return period. Rows show seven gauges (03378500: Wabash River at New Harmony, Illinois; 03381500: Little Wabash River at Carmi, Illinois; 03381700: Ohio River at Old Shawnetown, Kentucky-Illinois; 03399800: Ohio River at Smithland Dam, Kentucky; 03612600: Ohio River at Olmstead, Illinois; 07010000: Mississippi River at St Louis, Missouri, and 07022000: Mississippi River at Thebes, Illinois). Left column shows the transect measurements of stage and streamflow with a fitted Loess curve (blue line) and confidence intervals (red dashed lines), along with the estimated streamflow value at flood stage ("bankfull channel capacity", orange*

circle) with an empirically estimated two-year exceedance flow (blue circle). Middle column shows channel capacity values over time, estimated by adding the residuals of the stage-discharge curve to the estimated bankfull discharge, following Slater et al. (2015) (ref. 1). The first and last values of channel capacity (red circles) are extracted from the fitted linear regression (blue line). Regression statistics are indicated at the top of the panel. Right column shows the empirically estimated return periods associated with channel capacity at the start and end of the record, with associated dates. Grey circles indicate flood peaks extracted over the period of record and their corresponding return period (see Methods).

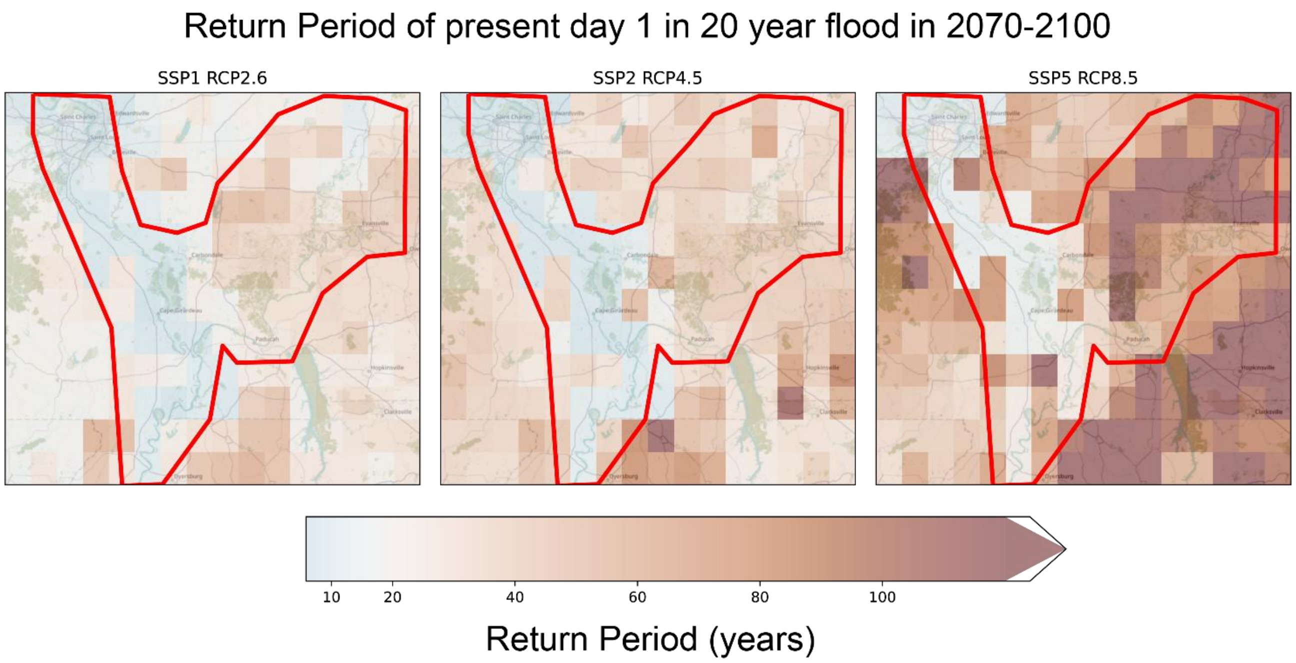

Supplementary Figure 2: *The return period of a present day 20-year flood event in 2071-2100 using 3 climate scenarios. Data based on flood model projections forced using the median of CMIP6 simulations as given in Hirabayashi et al (2021; ref. 2) (see Methods). The study area is bounded by the red lined polygon. Basemap data © OpenStreetMap contributors.*

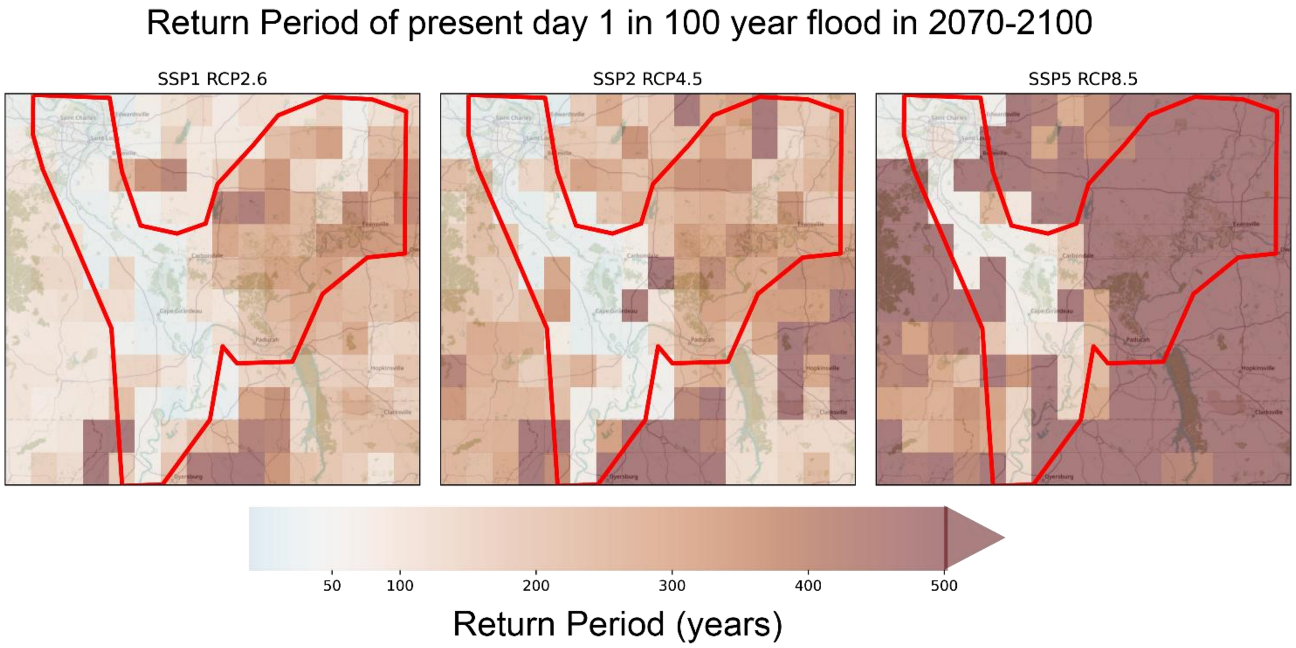

Supplementary Figure 3: *The return period of a present day 100-year flood event in 2071-2100 using 3 climate scenarios. Data based on flood model projections forced using the median of CMIP6 simulations as given in Hirabayashi et al (2021; ref. 2) (see Methods). The study area is bounded by the red lined polygon. Basemap data © OpenStreetMap contributors.*

Supplementary Table 1: *Annual Exceedance Probabilities (AEP) for the 2011 flood event as calculated by Driscoll et al (2014; ref. 39) at six gauging stations (Station ID locations are shown in Figure 1 of the main paper) located within the model domain. To estimate an overall AEP for the 2011 flood event, we take the mean values of the AEP for each of these six gauges. We therefore find an AEP of 1.2 with a 95% confidence interval of 0.75 and 3.85 for the 2011 flood. These AEP values correspond to ~1 in 80-year return period flood, with a 95% confidence interval ranging from ~1 in 25-year to ~1 in 130-year return periods. Note that gauges 3378500 and 3381700 have very large flows, with the AEP appearing to be ~0.1 as indicated by the growth curves from the stations.*

Gauge ID	Gauge Name	AEP	AEP 95%	AEP 5%
3377500	Wabash River at Mount Carmel, Illinois	2.1	0.4	4
3378500	Wabash River at New Harmony, Indiana	0.1	0.1	0.1
3381500	Little Wabash River at Carmi, Illinois	0.7	0.3	2.5
3381700	Ohio River at Old Shawneetown, Illinois	0.1	0.1	0.1
3399800	Ohio River at Smithland Dam, Smithland, Kentucky	0.2	1	9.5
3611500	Ohio River at Metropolis, Illinois	4	2.6	6.9
**Mean Values**		**1.2**	**0.75**	**3.85**

Supplementary Table 2: *Model skill scores for a range of commonly employed metrics (see Methods) as obtained for the simulated 1 in 75-year flood across the entire model domain and for a range of simulated bankfull return period values.*

Simulation Bankfull Return Period (BFRP)	Critical Success Index (CSI)	Hit Rate (HR)	Miss Rate (MR)	False Alarm Ratio (FAR)
1.1	0.584	0.988	0.012	0.412
1.5	0.59	0.967	0.033	0.398
2	0.595	0.96	0.038	0.378
5	0.602	0.902	0.098	0.356
10	0.592	0.855	0.145	0.342

Supplementary Table 3. *Simulated inundated areas and estimated people exposed for the model scenarios illustrated in Figure 4. These scenarios comprise: (1) a baseline of present-day climate and a bankfull channel conveyance capacity represented by the 2-year bankfull return period (BFRP) flow; (2) present-day climate with a BFRP at the upper bound (1.1 yrs) of present-day values estimated from the gauges in the study area; (3) present-day climate with a BFRP at the lower bound (0.3 years) of present-day values estimated from the gauges in the study area; (4) present-day climate with a BFRP set at the upper bound (2.1 years) of historical values estimated from gauges in the study area; (5) present-day climate with a BFRP set at the lower bound (1.2 years) of historical values estimated from gauges in the study area; (6) Future (2071-2100) climate change scenario (SSP 1 RCP 2.6) with a 2-year BFRP flow; (7) Future (2071-2100) climate change scenario (SSP 2 RCP 4.5) with a 2-year BFRP flow, and; (8) Future (2071-2100) climate change scenario (SSP5 RCP8.5) with a 2-year BFRP flow. Note that for the future climate scenarios, the present-day return periods of the two forcing flows are adjusted using change factors (Supplementary Figures 2 and 3).*

**Climate-Change and Channel Conveyance Scenarios**	**Forcing Flow Scenario**					
	**Relatively Frequent Flood (5-year RP)**		**Moderate Flood (20-year RP)**		**Large Flood (100-year RP)**	
	**Simulated Flood Extent (km^2)**	**Change in Simulated Flood Extent (%)**	**Simulated Flood Extent (km^2)**	**Change in Simulated Flood Extent (%)**	**Simulated Flood Extent (km^2)**	**Change in Simulated Flood Extent (%)**
1. Present-day climate and 2-yr BFRP (baseline)	6,220		10,204	-	13,781	-
2. Present-day climate and 1.1-yr (upper observed) BFRP	9,306	49.6	12,289	20.4	15,058	9.3
3. Present-day climate and (lower observed) 0.3-yr BFRP	15,654	151.6	14,506	42.2	16,697	21.2
4. Present-day climate and 2.1-yr (upper historical) BFRP	5,509	-11.6	10,290	-0.1	13,752	-0.3
5. Present-day climate and 1.2-yr (lower historical) BFRP	8,864	42.5	12,020	17.8	14,884	8.0
6. Future (2071-2100) climate (SSP1 RCP2.6) and 2-year BFRP	-	-	9,288	-9.0	13,702	-0.6
7. Future (2071-2100) climate (SSP2 RCP4.5) and 2-year BFRP	-	-	9,864	-3.3	13,964	1.3
8. Future (2071-2100) climate (SSP5 RCP8.5) and 2-year BFRP	-	-	12,630	23.8	15,177	10.1
			**Population Exposed**	**Change in Population Exposed (%)**	**Population Exposed**	**Change in Population Exposed (%)**
1. Present-day climate and 2-yr BFRP (baseline)	41,956		119,059	-	205,817	-
2. Present-day climate and 1.1-yr (upper observed) BFRP	104,588	149.2	181,283	52.3	237,162	15.2

3. Present-day climate and (lower observed) 0.3-yr BFRP	197,865	471.6	278,244	133.7	273,296	32.8
4. Present-day climate and 2.1-yr (upper historical) BFRP	49,272	17.4	118,571	-0.4	206,630	-0.4
5. Present-day climate and 1.2-yr (lower historical) BFRP	93,832	223.6	172,358	44.8	232,732	13.1
6. Future (2071-2100) climate (SSP1 RCP2.6) and 2-year BFRP	-	-	60,615	-49.1	175,595	-14.7
7. Future (2071-2100) climate (SSP2 RCP4.5) and 2-year BFRP	-	-	63,204	-46.9	177,770	-13.6
8. Future (2071-2100) climate (SSP5 RCP8.5) and 2-year BFRP	-	-	115,177	-3.3	245,801	19.4

73

Supplementary Table 4 *Calculations of time-evolution of flood hazard (inundation extent) and impact (populations exposed) for the relatively frequent (5-year) driving flood discharge and as forced by observed historical changes in channel conveyance capacity versus future climate changes. The numbered scenarios are defined in the caption to Supplementary Table 3.*

**Scenario**	**Flood Extent Calculations**			**Population Exposure Calculations**		
	**Simulated Flood Extent (km^2)**	**Change in Simulated Flood Extent (km^2)**	**Rate of Change of Simulated Flood Extent (km^2/decade)**	**People Exposed**	**Change in People Exposed**	**Rate of Change of Exposure (people/decade)**
1. Present-day climate and 2-yr BFRP (baseline)	6,220	n/a	n/a	41,956	n/a	n/a
4. Start point of channel change scenario using present-day climate and 2.1-year (historical) BFRP	5,509	n/a	n/a	49,272	n/a	n/a
5. End point of channel change scenario using present day climate and 1.2-year (historical) BFRP	8,864	+3,355[1]	**+781[3]**	93,832	+44,560[1]	**+10,374[3]**

Notes on calculations:

1. computed using difference between scenario 5 and scenario 4.

Supplementary Table 5 *Calculations of time-evolution of flood hazard (inundation extent) and impact (populations exposed) for the moderate (20-year) driving flood discharge and as forced by observed historical changes in channel conveyance capacity versus future climate changes. The numbered scenarios are defined in the caption to Supplementary Table 3.*

Scenario	Flood Extent Calculations			Population Exposure Calculations		
	**Simulated Flood Extent (km^2)**	**Change in Simulated Flood Extent (km^2)**	**Rate of Change of Simulated Flood Extent (km^2/decade)**	**People Exposed**	**Change in People Exposed**	**Rate of Change of Exposure (people/decade)**
1. Present-day climate and 2-yr BFRP (baseline)	10,204	n/a	n/a	119,059	n/a	n/a
4. Start point of channel change scenario using present-day climate and 2.1-year (historical) BFRP	10,290	n/a	n/a	118,571	n/a	n/a
5. End point of channel change scenario using present day climate and 1.2-year (historical) BFRP	12,020	+1,730[1]	**+403[3]**	172,358	+53,787[1]	**+12,523[3]**
6. Future (2071-2100) climate (SSP1 RCP2.6) and 2-year BFRP	9,288	-916[2]	**-150[4]**	60,615	-58,444[2]	**-9,741[4]**
7. Future (2071-2100) climate (SSP2 RCP4.5) and 2-year BFRP	9,864	-340[2]	**-56[4]**	63,204	-55,855[2]	**-9,309[4]**
8. Future (2071-2100) climate (SSP5 RCP8.5) and 2-year BFRP	12,630	+2,426[2]	**+404[4]**	115,177	-3,882[2]	**-647[4]**

Notes on calculations:

1. computed using difference between scenario 5 and scenario 4.
2. computed using difference between this scenario and the baseline scenario 1.

3. computed by dividing the estimated change (reported in cell to left) by the 43-year period of gauge record, and then converting the annual rate so-obtained to a decadal rate.
4. computed by dividing the estimate change (reported in cell to left) by the 60-year period between the present-day (2025) and the mid-point (2085) of the future climate, and then converting the annual rate so obtained to a decadal rate.

Supplementary References

1. Slater, L. J., Singer, M.B. and Kirchner, J.W., 2015. Hydrologic versus geomorphic drivers of trends in flood hazard, Geophys. Res. Lett., 42, 1–7. DOI:10.1002/2014GL062482
2. Hirabayashi, Y., Tanoue, M., Sasaki, O. et al. 2021. Global exposure to flooding from the new CMIP6 climate model projections. Scientific Reports, 11, 3740. DOI: 10.1038/s41598-021-83279-w

Supplementary Table 6 *Calculations of time-evolution of flood hazard (inundation extent) and impact (populations exposed) for the large (100-year) driving flood discharge and as forced by observed historical changes in channel conveyance capacity versus future climate changes. The numbered scenarios are defined in the caption to Supplementary Table 3.*

**Scenario**	**Flood Extent Calculations**			**Population Exposure Calculations**		
	**Simulated Flood Extent (km^2)**	**Change in Simulated Flood Extent (km^2)**	**Rate of Change of Simulated Flood Extent (km^2/decade)**	**People Exposed**	**Change in People Exposed**	**Rate of Change of Exposure (people/decade)**
1. Present-day climate and 2-yr BFRP (baseline)	13,781	n/a	n/a	205,817	n/a	n/a
4. Start point of channel change scenario using present-day climate and 2.1-year (historical) BFRP	13,752	n/a	n/a	206,630	n/a	n/a
5. End point of channel change scenario using present day climate and 1.2-year (historical) BFRP	14,884	+1,127[1]	**+262**[3]	232,732	+26,102[1]	**+6,077**[3]
6. Future (2071-2100) climate (SSP1 RCP2.6) and 2-year BFRP	13,702	-79[2]	**-13**[4]	175,595	-30,222[2]	**-5,037**[4]
7. Future (2071-2100) climate (SSP2 RCP4.5) and 2-year BFRP	13,964	+183[2]	**+31**[4]	177,770	-28,047[2]	**-4,675**[4]
8. Future (2071-2100) climate (SSP5 RCP8.5) and 2-year BFRP	15,177	+1,396[2]	**+233**[4]	245,801	+39,984[2]	**+6,664**[4]

Notes on calculations:

1. computed using difference between scenario 5 and scenario 4.
2. computed using difference between this scenario and the baseline scenario 1.

3. computed by dividing the estimated change (reported in cell to left) by the 43-year period of gauge record, and then converting the annual rate so-obtained to a decadal rate.
4. computed by dividing the estimate change (reported in cell to left) by the 60-year period between the present-day (2025) and the mid-point (2085) of the future climate, and then converting the annual rate so obtained to a decadal rate.
