# Supplementary material for: River channel change can affect flood hazard and impact substantially
Source: Commun Earth Environ. 2026 May 5;7(1):585. doi: 10.1038/s43247-026-03517-9 (PMC13349851; doi:10.1038/s43247-026-03517-9)
Supplement: Supplementary file 1 — Transparent Peer Review file [file 43247_2026_3517_MOESM1_ESM.pdf]

nature portfolio

Peer Review File

River Channel Change Can Affect Flood Hazard and Impact Substantially

Corresponding Author: Dr Laurence Hawker

This file contains all editorial decision letters in order by version, followed by all author rebuttals in order by version.

Attachments originally included by the reviewers as part of their assessment can be found at the end of this file.

Version 0:

Decision Letter:

** Please ensure you delete the link to your author home page in this e-mail if you wish to forward it to your coauthors **

Dear Dr Hawker,

Your manuscript titled "River Channel Change Can Affect Flood Hazard and Risk as Much as Climate" has now been seen by 3 reviewers, whose comments are appended below. You will see that they find your work of some potential interest. However, they have raised quite substantial concerns that must be addressed. In light of these comments, we cannot accept the manuscript for publication, but would be interested in considering a revised version that fully addresses these serious concerns. Specifically, a revised manuscript must:

*** Investigate additional flood return periods (e.g., 2-, 5-, 10-, and 500-year) to assess how different flood magnitudes interact with river channel conveyance capacity and affect flood hazard estimates.

*** Address uncertainties in channel conveyance capacity (depth, width, roughness), return period flow estimations, and sensitivity analyses.

*** Clearly differentiate the effects of river channel changes from land-use changes, sedimentation, and dam operations, and discuss their role in flood hazard assessments.

We hope you will find the reviewers' comments useful as you decide how to proceed. Should additional work allow you to address these criticisms, we would be happy to look at a substantially revised manuscript. If you choose to take up this option, please either highlight all changes in the manuscript text file, or provide a list of the changes to the manuscript with your responses to the reviewers.

When resubmitting, please provide a point-by-point response to the reviewers' comments. Please submit your responses as a separate file, distinct from your cover letter where you can add responses to the Editors' comments that you do not want to be made available to the reviewers. Word files are preferred. We recommend that any figures, tables or graphs that are included in the response to reviewers are also included in the main article or Supplementary Information.

Please bear in mind that we will be reluctant to approach the reviewers again in the absence of substantial revisions.

If the revision process takes significantly longer than three months, we will be happy to reconsider your paper at a later date, as long as nothing similar has been accepted for publication at Communications Earth & Environment or published elsewhere in the meantime.

We are committed to providing a fair and constructive peer-review process. Please do not hesitate to contact us if you wish to discuss the revision in more detail.

Please use the following link to submit your revised manuscript, point-by-point response to the reviewers' comments with a list of your changes to the manuscript text (which should be in a separate document to any cover letter), a tracked-changes version of the manuscript (as a PDF file) and any completed checklist:

Link Redacted

** This url links to your confidential home page and associated information about manuscripts you may have submitted or be reviewing for us. If you wish to forward this email to co-authors, please delete the link to your homepage first **

Please do not hesitate to contact us if you have any questions or would like to discuss the required revisions further. Thank you for the opportunity to review your work.

Best regards,

Rahim Barzegar, PhD
Editorial Board Member
Communications Earth & Environment
orcid.org/0000-0002-1941-2991

Alireza Bahadori, PhD
Associate Editor
Communications Earth & Environment
Consulting Editor, Communications Sustainability

EDITORIAL POLICIES AND FORMAT

If you decide to resubmit your paper, please ensure that your manuscript complies with our editorial policies and complete and upload the checklist below as a Related Manuscript file type with the revised article:

Editorial Policy Policy requirements
(Download the link to your computer as a PDF.)

For Manuscripts that fall into the following fields:
• Behavioural and social science
• Ecological, evolutionary & environmental sciences
• Life sciences
An updated and completed version of our Reporting Summary must be uploaded with the revised manuscript
You can download the form here:
https://www.nature.com/documents/nr-reporting-summary.zip

For your information, you can find some guidance regarding format requirements summarized on the following checklist: (https://www.nature.com/documents/commsj-phys-style-formatting-checklist-article.pdf) and formatting guide (https://www.nature.com/documents/commsj-phys-style-formatting-guide-accept.pdf).

REVIEWER COMMENTS:

Reviewer #1 (Remarks to the Author):

The paper deals with a very relevant issue, that is how river channel change may affect flood hazard assessment. Since river channel changes may affect remarkably channel conveyance capacity, this is something that all hydraulic models should take into consideration. That said, I have some concerns about this paper.
1. Are Global Flood Models the suitable models to address this issue (i.e. how river channel change affect flood hazard)? I am quite sceptical about this. The GFMs were developed to be applied in data scarce regions, and it is widely recognized that GFMs have some limitation (see for instance Sampson et al., 2015, WRR, cited in this work). It seems to me that it could be more useful to address the issue at local scale (i.e. by high resolution modelling), before addressing the issue with GFMs that still suffered (and likely will suffered for many years) in the definition of channel geometry (specifically in the definition of channel depth).
2. Following the previous point, I am wondering how much novelty comes out from this paper. The authors point out the weakness of GFMs in representing river channels (see L 42-44), but, again, this is well known. Therefore, I do not find so relevant the first key outcome of the paper (L 193-196): it is not a surprising outcome considering that GFMs define channel geometry using bankfull discharge, rather than by real morphological measurements.

It seems to me that some major changes should be carried out, including re-definition of the paper aims.
1. It would be worth to clarify that river channel changes are crucial in general for flood assessment, not only for GFMs.
2. A brief description of what GFMs are (e.g. why such models were developed, potential and limitations) and differences with other hydraulic models (e.g. high-resolution local models) is needed.
3. If the main aim is to point out the large uncertainty that still exists in these models, this is fine for a more appropriate use of

GFMs (and this could be probably enough to justify publication of this work). I would be more cautious about comparison between effects of channel changes and climate changes. The paper is based on a single case study, and this is acknowledged (L209-210), but my suspect is that channel changes effects could even much larger of what shown in this work. The authors should consider that cross sections at gauging station are, in general, more stable than other sections along rivers.
4. Last, but not least, it would be useful some considerations about future perspectives of GFMs (L 218-219 are not enough). How much channel geometry could be improved in GFMs in the near future? Data to improve the definition of channel width are already available, but channel depth (i.e. bathymetry) will be likely remain an issue for some years.

Reviewer #2 (Remarks to the Author):

Review for the manuscript titled- "River Channel Change Can Affect Flood Hazard and Risk as Much as Climate"
Decision: Accept with minor to moderate revisions

This paper investigates the role of river channel morphology changes in influencing flood hazard and risk, comparing its impact to that of climate change. The authors employ the Fathom Global Flood Model (GFM) to assess flood extents and population exposure under various channel conveyance scenarios and different return periods. The authors demonstrate that neglecting channel morphological changes in GFMs can lead to substantial underestimations of flood hazard and risk. They further argue that historical river channel evolution has exerted an influence comparable to, or exceeding, that of climate-driven changes projected for the future.
Overall, the presentations of the data, methodology, results and writing are great. This is an excellent and impactful paper that challenges conventional flood modeling assumptions. The results are well-supported by empirical evidence and robust modeling techniques. However, addressing the concerns outlined below would significantly strengthen the study’s global applicability.
I have some minor comments and suggestions. Following are the comments and suggestions:
• The study challenges a long-standing assumption in GFMs—that the bankfull return period is a fixed 2-year RP. While the authors assert that their findings are globally applicable, the study focuses exclusively on the Mississippi River system. Providing additional evidence from other major river systems to demonstrate the broader relevance of these findings can be beneficial.
• I understand this may be out of scope for this study, however I would be very much interested to see studies on morphological changes in other global river basins (e.g., Amazon, Ganges, or Mekong) to reinforce the claim that fixed 2-year RPs are unrealistic worldwide.
• The study primarily investigates 20-year and 100-year return period floods. What are your thoughts on more frequent (2, 5 or 10 year) and more impactful (500-year) floods, which might interact differently with river channel conveyance capacities.
• You have used CMIP6-derived changes in return periods under different climate scenarios. These projections do not account for factors such as land-use change, sedimentation, and manmade interventions, which can further alter river channel conveyance. At least for the years 1980 and 2023. How can you differentiate these factors from climate change? Some additional discuss how factors beyond climate change (e.g., deforestation, urbanization, dam operations) could further impact flood hazard estimates would benefit the novelty of the study.
• While using WorldPop to calculate the exposure there is no mention of the temporal information on the data.
• Figure 1: The arrows show the trend in Channel conveyance, however instead of referring to Figure 2 adding what are the different direction of the arrow means would help readers to understand better.
• LN 62: what is “c.” here?
• LN 224: in the Fathom model, how is the roughness represented?

Reviewer #3 (Remarks to the Author):

The study investigates a very common assumption in many GFMs that the river bankfull conveyance capacity corresponds to a 2-year return flow. For a case study of a part of the Mississippi River, the actual flow corresponding to bankfull conveyance capacity was determined as well as its temporal change. Furthermore, a sensitivity analysis was employed to compare changes in flooding from temporal changes in bankfull conveyance flow and climate change. The study found significant variation of the bankfull return flow from the often-used 2-year assumption. It also found that changes in bankfull return flow are similar to changes in projected climate change with respect to the flood hazard.
This is an important study highlighting the need to better understand and incorporate river bathymetry in GFMs.
The study is well written and uses appropriate methods. However, some key assumptions are not well motivated and uncertainties throughout the experiment are not well discussed in relation to the final conclusions as detailed below. I therefore suggest minor revision to address these points.

Main comments
1. In many places, you discuss channel conveyance capacity which is determined by the cross-sectional shape and roughness of the channel bed. However, in the sensitivity analysis, you seem to only include the depth. It's also not clear from the methods how the channel width and Manning roughness in the model are parametrized. Can you clarify in the manuscript what you mean by channel conveyance capacity, motivate the choice to focus on depth only, and discuss how changes in depth and roughness could affect the results?

2. Your model is based on flows estimated from an RFFA with "6% and 29% error for the 10-year and 100-year flows". A systematic bias in the estimated return flows from the RFFA can have a significant influence on your results. Can you report the specific errors at the gauge stations in the area and if these are significant, address the effect of these biases on the results?

3. Uncertainties in many steps of the experiment are not accounted for or discussed in relation to the conclusions. I suggest discussing and where possible including the following uncertainties in your figures: uncertainties in the RP flows (see previous point), uncertainties in the extrapolation of the sensitivity curves (figure 4), uncertainties in the estimation of temporal changes in BFRP and the range of temporal changes in BFRP between locations, and uncertainties in changes in RP due to climate change.

4. In many places, you use the term flood risk as the population exposed for a certain return period. However, for flood risk, the impacts have to be assessed under a range of exceedance probabilities. I suggest the authors use the term flood impact instead to be more consistent with the literature.

Minor comments
L17: It is unclear what the range in underestimation of flood extents and exposed population represents. It seems related to 20 and 100-year return periods at different estimated BFRP. Can you clarify this in the text such that readers can better interpret these numbers.

L146 (Figure 4): While care has been taken to select a model with good fit, there is (large) statistical uncertainty in extrapolating predictions of five points. This uncertainty should be shown in the figure.

L159: You state that the relative effects of temporal changes in conveyance and climate change on flood inundation are similar in magnitude. For this conclusion, you take the most extreme change in estimated BFRP from all gauges. Can you motivate this choice, or include a range of measured changes across all gauges in your results?

L267: Can you clarify why the actual observed 2011 flow was not used for validation instead of the 75-year return period flow? Since there are large spatial differences in AEP as shown in extended Data Table 1, how do you make sure you're not comparing apples and pears?

L327: You assume stationary flow return periods over the period of 1980 until present and use these to estimate the BFRP. Can you show that this is in fact an acceptable assumption for the stations assessed?

L335: You use changes in RPs from Hirabayashi et al. (2021) to reflect climate change in your study. However, the baseline for their study (1971-2000) is different from the baseline of your study (1980-2023). Can you discuss how this difference may affect your results and conclusions?

L339: I suggest renaming this section to "extrapolation of flood predictors" which is more descriptive of the content as you also fit different curves in other sections of the methodology.

L363: You use the multi-model median, but how did you deal with the spatial distribution of changes in return period flow as shown in Extended Data Figure 2-3.

Reproducibility: Some manual data filtering was done for the streamflow transect data (L301) and no scripts seem to be provided to reproduce the analysis. It would be strongly recommended to share the (filtered) preprocessed data if possible as well as the code to make the research reproducible.

** Visit Nature Research's author and referees' website at www.nature.com/authors for information about policies, services and author benefits**

Communications Earth & Environment is committed to improving transparency in authorship. As part of our efforts in this direction, we are now requesting that all authors identified as ‘corresponding author’ create and link their Open Researcher and Contributor Identifier (ORCID) with their account on the Manuscript Tracking System prior to acceptance. ORCID helps the scientific community achieve unambiguous attribution of all scholarly contributions. You can create and link your ORCID from the home page of the Manuscript Tracking System by clicking on ‘Modify my Springer Nature account’ and following the instructions in the link below. Please also inform all co-authors that they can add their ORCIDs to their accounts and that they must do so prior to acceptance.
https://www.springernature.com/gp/researchers/orcid/orcid-for-nature-research

For more information please visit http://www.springernature.com/orcid

If you experience problems in linking your ORCID, please contact the Platform

Support Helpdesk.

Version 1:

Decision Letter:

<*** REMEMBER TO ATTACH REVISIONS CHECKLIST (WORD)***>

** Please ensure you delete the link to your author home page in this e-mail if you wish to forward it to your coauthors **

Dear Dr Hawker,

Your revised manuscript titled "River Channel Change Can Affect Flood Hazard and Impact Substantially" has now been seen by our reviewers, whose comments appear below. In light of their advice we are delighted to say that we are happy, in principle, to publish a suitably revised version in Communications Earth & Environment.

We therefore invite you to revise your paper one last time to address the remaining concerns of our reviewers. At the same time we ask that you edit your manuscript to comply with our format requirements and to maximise the accessibility and therefore the impact of your work.

EDITORIAL REQUESTS:

Please review our specific editorial comments and requests regarding your manuscript in the attached "Editorial Requests Table".

*****Please take care to match our formatting and policy requirements. We will check revised manuscript and return manuscripts that do not comply. Such requests will lead to delays. *****

Please outline your response to each request in the right hand column. Please upload the completed table with your manuscript files as a Related Manuscript file.

If you have any questions or concerns about any of our requests, please do not hesitate to contact me.

SUBMISSION INFORMATION:

In order to accept your paper, we require the files listed at the end of the Editorial Requests Table; the list of required files is also available at https://www.nature.com/documents/commsj-file-checklist.pdf .

OPEN ACCESS:

Communications Earth & Environment is a fully open access journal. Articles are made freely accessible on publication. For further information about article processing charges, open access funding, and advice and support from Nature Portfolio, please visit https://www.nature.com/commsenv/open-access

At acceptance, you will be provided with instructions for completing the open access licence agreement on behalf of all authors. This grants us the necessary permissions to publish your paper. Additionally, you will be asked to declare that all required third party permissions have been obtained, and to provide billing information in order to pay the article-processing charge (APC).

Please use the following link to submit the above items:
Link Redacted
** This url links to your confidential home page and associated information about manuscripts you may have submitted or be reviewing for us. If you wish to forward this email to co-authors, please delete the link to your homepage first **

We hope to hear from you within two weeks; please let us know if you need more time.

Best regards,

Rahim Barzegar, PhD
Editorial Board Member
Communications Earth & Environment
orcid.org/0000-0002-1941-2991

Alireza Bahadori, PhD
Senior Editor
Communications Earth & Environment

Consulting Editor
Communications Sustainability

REVIEWERS' COMMENTS:

Reviewer #1 (Remarks to the Author):

I think that the authors addressed very well the points that I raised in my first review of this manuscript (as well as the points/suggestions of the other two reviewers). Overall, I am happy with the new version of the paper. The aims and novelties of the paper are clear and the outcomes are of interest for a large audience.
Nicola Surian

Reviewer #3 (Remarks to the Author):

The authors have substantially strengthened the manuscript in this revision. In particular, the improved discussion of uncertainties in the approach enhances the robustness and transparency of the analysis. The authors have also done a good job of better positioning their findings. At this stage, only a few minor comments remain that, once addressed, will further refine the clarity and presentation.

L15. I suggest clarifying that your findings are based on seven gauges in a case study of the Mississippi floodplain.

L152. The limitation of the Fathom model in representing bankfull return periods (BFRP) below 1.1 years due to its reliance on annual maxima for the RFFA is clear. However, for this experiment the model could have been based on peaks-over-threshold (POT) extreme values from local gauges to overcome this limitation and work around the constraints of the global Fathom model. Please provide reasoning for why you decided to use the RFFA and extrapolate the flood predictors from only five data points instead. In addition, if the actual BFRP is often found to be smaller than 1.1 years, please add a discussion on how to incorporate this in large-scale flood models using RFFA based on annual maxima going forward.

** Visit Nature Portfolio's author and referees' website at www.nature.com/authors for information about policies, services and author benefits**

Open Access This Peer Review File is licensed under a Creative Commons Attribution 4.0 International License, which permits use, sharing, adaptation, distribution and reproduction in any medium or format, as long as you give appropriate credit to the original author(s) and the source, provide a link to the Creative Commons license, and indicate if changes were

made.
In cases where reviewers are anonymous, credit should be given to 'Anonymous Referee' and the source.
The images or other third party material in this Peer Review File are included in the article's Creative Commons license, unless indicated otherwise in a credit line to the material. If material is not included in the article's Creative Commons license and your intended use is not permitted by statutory regulation or exceeds the permitted use, you will need to obtain permission directly from the copyright holder.
To view a copy of this license, visit https://creativecommons.org/licenses/by/4.0/

Dear Editors & Reviewers,

We appreciate you constructive comments and your time in making them. We have responded to them below. Thank you for your patience while we made these revisions as we had to deal with a number of challenges.

Kind Regards

Author Team

EDITOR COMMENTS:
Your manuscript titled "River Channel Change Can Affect Flood Hazard and Risk as Much as Climate" has now been seen by 3 reviewers, whose comments are appended below. You will see that they find your work of some potential interest. However, they have raised quite substantial concerns that must be addressed. In light of these comments, we cannot accept the manuscript for publication, but would be interested in considering a revised version that fully addresses these serious concerns.
We thank the editor for the opportunity to address the concerns raised. Below, we provide a detailed, point-by-point response, including clarification of the three issues you have identified, and we provide an outline of the changes made to the manuscript in light of these comments.

Specifically, a revised manuscript must:
1) Investigate additional flood return periods (e.g., 2-, 5-, 10-, and 500-year) to assess how different flood magnitudes interact with river channel conveyance capacity and affect flood hazard estimates.
This issue was raised by Referee 2 as a minor point. We outline how we have revised the manuscript to fully address the concern in our response R2-3, below.

2) Address uncertainties in channel conveyance capacity (depth, width, roughness), return period flow estimations, and sensitivity analyses.
This point was raised by Referee 3 and our response R3-3 (below) details how we have revised our paper to ensure that the concern is fully allayed.

3) Clearly differentiate the effects of river channel changes from land-use changes, sedimentation, and dam operations, and discuss their role in flood hazard assessments.
This issue was raised by Referee 2, who framed it as a minor point. We explain how we have revised the paper accordingly in our response R2-4 (below).

REVIEWER COMMENTS:
Reviewer #1 (Remarks to the Author):
The paper deals with a very relevant issue, that is how river channel change may affect flood hazard assessment. Since river channel changes may affect remarkably channel conveyance capacity, this is something that all hydraulic models should take into consideration. That said, I have some concerns about this paper.

R1-1. Are Global Flood Models the suitable models to address this issue (i.e. how river channel change affect flood hazard)? I am quite sceptical about this. The GFMs were developed to be applied in data scarce regions, and it is widely recognized that GFMs have some limitation (see for instance Sampson et al., 2015, WRR, cited in this work). It seems to me that it could be more useful to address the issue at local scale (i.e. by high resolution modelling), before addressing the issue with GFMs that still suffered (and likely will suffered for many years) in the definition of channel geometry (specifically in the definition of channel depth).
We thank the reviewer for raising this important point regarding the suitability and limitations of Global Flood Models (GFMs). We agree that GFMs were originally designed for applications in data-scarce regions and that their representation of channel geometry, particularly channel depth, is a recognised source of uncertainty. We also agree that high-resolution, reach-scale, modelling plays a crucial role in understanding detailed hydraulic responses to geomorphic change. Importantly, the model used in this study is not a global-scale GFM in the traditional sense. It is a regional GFM forming the backbone of a widely published and used U.S.-wide hazard analysis. Our intention is not to claim that GFMs are the optimal tools for process-level hydraulic diagnosis, but rather to assess how their structural assumptions influence large-scale hazard estimates. To ensure this framing is clear, we highlight the purpose of our study (L66-68 of revised text) and also explicitly clarify the regional nature of the GFM applied here (see revised text at L70-72).

R1-2. Following the previous point, I am wondering how much novelty comes out from this paper. The authors point out the weakness of GFMs in representing river channels (see L 42-44), but, again, this is well known. Therefore, I do not find so relevant the first key outcome of the paper (L 193-196): it is not a surprising outcome considering that GFMs define channel geometry using bankfull discharge, rather than by real morphological measurements.
As noted above, we agree that the generic limitation of GFMs in representing channel geometry is well established. However, our paper does not aim to restate this. Instead, the novelty lies in quantifying, for the first time, how dynamic channel change propagates into flood hazard estimates within an operational, large-scale, flood model. Specifically:

- No previous study has empirically quantified the magnitude or spatial distribution of flood-hazard biases arising from spatial variations in channel capacity and channel evolution across a large domain. While it is known that channel geometries in GFMs are simplified, the functional consequences of real-world channel change on hazard outputs have not been measured. Our results demonstrate how this known structural limitation manifests in practice, something that cannot be inferred from model design alone. This aspect is clarified at L54-56 in the revised text.
- The direction and scale of the impacts are not predictable from first principles. Although one might expect errors, our analysis shows that hazard vary depending on local geomorphic context. This emergent behaviour has not been characterised previously and is directly relevant for users of large-scale hazard products. We now emphasize this point at L55-57 in the revised text.
- The model used here is the regional GFM forming the backbone of U.S.-wide flood hazard assessment (which we have now clarified as per our response to R1-1, above). Therefore, the novelty is not in identifying a known model weakness, but in assessing its real-world

implications for the exact modelling system used for national risk estimation, regulation, and re/insurance exposure modelling.
- In our discussion we clarify that our findings inform how channel evolution introduces material impact-relevant biases (L232-249 in the revised manuscript).

R1-3. It would be worth to clarify that river channel changes are crucial in general for flood assessment, not only for GFMs.
We agree that river channel change is a fundamental aspect of flood hazard assessment across all modelling scales and approaches, not only within Global Flood Models (GFMs). Channel evolution affects conveyance capacity, flow routing, inundation patterns, and therefore hazard estimates in both local high-resolution hydraulic models and large-scale models. In response to these challenges we have more clearly framed our rationale to reflect this context: namely, channel change is universally important for flood modelling, but the consequences of ignoring channel change have never been quantified within the operational, continental-scale GFM framework currently used for U.S. national hazard assessment.
As noted above (R1-2) we have made changes to address the reviewer's point and enhance the clarity of the manuscript's aims.

R1-4. A brief description of what GFMs are (e.g. why such models were developed, potential and limitations) and differences with other hydraulic models (e.g. high-resolution local models) is needed.
We thank the reviewer for this constructive suggestion. We have added a brief description at L38-43 accordingly.

R1-5. If the main aim is to point out the large uncertainty that still exists in these models, this is fine for a more appropriate use of GFMs (and this could be probably enough to justify publication of this work). I would be more cautious about comparison between effects of channel changes and climate changes. The paper is based on a single case study, and this is acknowledged (L209-210), but my suspect is that channel changes effects could even much larger of what shown in this work. The authors should consider that cross sections at gauging station are, in general, more stable than other sections along rivers.
We thank the reviewer for this helpful suggestion. We agree that the comparison with climate-change effects should be treated cautiously. Nevertheless, we believe positioning the results at this site within the context of climate change has substantial contextual value and helps to frame our findings within the much larger body of work on climate change impacts.
In response, we have downplayed this comparison somewhat, and have removed climate change from the title. Additionally, we have substantially revised the text in the discussion (primarily at L182-199 of the revised text) to present it only as contextual illustration rather than a central conclusion. We also agree that cross sections at gauging stations tend to be more stable than the wider river network. We now explicitly acknowledge (L195-199 of the revised text) that this likely makes our estimates conservative and that channel-change effects elsewhere may be larger. We appreciate the reviewer's guidance on this, which has strengthened the clarity and focus of the manuscript.

R1-6. Last, but not least, it would be useful some considerations about future perspectives of GFMs (L218-219 are not enough). How much channel geometry could be improved in GFMs in the near future? Data to improve the definition of channel width are already available, but channel depth (i.e. bathymetry) will be likely remain an issue for some years.
We thank the reviewer for this helpful suggestion. We agree that a clearer discussion of future GFM capabilities could strengthen the paper and have added (see L261-265 of the revised manuscript) some sentences to offer a perspective on emerging GFM development opportunities.

Reviewer #2 (Remarks to the Author):
This paper investigates the role of river channel morphology changes in influencing flood hazard and risk, comparing its impact to that of climate change. The authors employ the Fathom Global Flood Model (GFM) to assess flood extents and population exposure under various channel conveyance scenarios and different return periods. The authors demonstrate that neglecting channel morphological changes in GFMs can lead to substantial underestimations of flood hazard and risk. They further argue that historical river channel evolution has exerted an influence comparable to, or exceeding, that of climate-driven changes projected for the future. Overall, the presentations of the data, methodology, results and writing are great. This is an excellent and impactful paper that challenges conventional flood modeling assumptions. The results are well-supported by empirical evidence and robust modeling techniques. However, addressing the concerns outlined below would significantly strengthen the study's global applicability.
I have some minor comments and suggestions. Following are the comments and suggestions:
We are delighted to receive such strong support for our paper and are particularly grateful for the comments on the impactful nature of our work.

R2-1. The study challenges a long-standing assumption in GFMs—that the bankfull return period is a fixed 2-year RP. While the authors assert that their findings are globally applicable, the study focuses exclusively on the Mississippi River system. Providing additional evidence from other major river systems to demonstrate the broader relevance of these findings can be beneficial.
We thank the reviewer for this helpful comment. While our empirical case study focuses on the Mississippi system, the broader point is indeed not limited to this basin. We have clarified this and now support the wider relevance of our findings with evidence from the literature. First, in terms of spatial changes and generalisability across systems, our recent work (e.g., Liu et al., 2024) based on gauge data has already shown that bankfull return periods vary substantially globally, demonstrating that the spatially-uniform 2-year assumption does not hold. This point is addressed specifically at L254-256 in the revised manuscript. In terms of evolution through time, Slater et al. (2015) show that bankfull return periods, across multiple U.S. gauging stations change over time - thus indicating that temporal variability is not unique to the Mississippi system that we use as a case study in this work (this point is now added at L256-258 of the revised paper). The available evidence therefore suggests that both spatial and temporal variability in BFRP are widespread.

R2-2. I understand this may be out of scope for this study, however I would be very much

interested to see studies on morphological changes in other global river basins (e.g., Amazon, Ganges, or Mekong) to reinforce the claim that fixed 2-year RPs are unrealistic worldwide.
As per our response to R2-1 (above) we also now note in the revised paper that new work does now provide the evidence of global variability in BFRPs that the reviewer is seeking. However, undertaking a global assessment of the effects of these variations in bankfull capacity is indeed beyond the scope of the present study, which is focused on providing a foundational proof-of-concept using the best long-term geomorphic records currently accessible. We have added text to the Discussion highlighting this as a key future research direction and noting that our results provide a basis for these broader, globally comparative analyses once new datasets mature. We appreciate the reviewer's forward-looking perspective and agree it will be an important avenue for future work, a point that we now make at L264-268 of the revised manuscript.

R2-3. The study primarily investigates 20-year and 100-year return period floods. What are your thoughts on more frequent (2, 5 or 10 year) and more impactful (500-year) floods, which might interact differently with river channel conveyance capacities.
We thank the reviewer for raising this question. More frequent events (2–10 year) are likely to be even more sensitive to channel conveyance, given that proportionately more of their flow will be conveyed in-bank flow as compared to rarer events, but they will also be less impactful in terms of risk. Very rare events (e.g., 500 year) will likely show reduced sensitivity once widespread floodplain inundation dominates. Therefore, we have added simulations for a relatively frequent 5-year event to explore the sensitivity to channel conveyance capacity for more frequent floods only, especially given the large computational burden of running multiple additional return periods. To clarify the rationale of our initial choices: the 20- and 100-year return periods are more commonly used for flood impact assessments and they were the selected return periods for the climate change simulations of Hirabayashi et al (2021). We do however appreciate your suggestion and by adding the 5-year event we have further highlighted the importance of sensitivity of models to channel conveyance capacity.

R2-4. You have used CMIP6-derived changes in return periods under different climate scenarios. These projections do not account for factors such as land-use change, sedimentation, and manmade interventions, which can further alter river channel conveyance. At least for the years 1980 and 2023. How can you differentiate these factors from climate change? Some additional discussion on how factors beyond climate change (e.g., deforestation, urbanization, dam operations) could further impact flood hazard estimates would benefit the novelty of the study.
We thank the reviewer for this helpful observation. We agree that factors such as land-use change, sedimentation, urbanisation, and dam operations can also influence channel conveyance and flood hazard. We have added a brief statement (L225-228) recognising these wider drivers, but it is not possible to model or isolate them within the scope of this study, which uses CMIP6 changes only as a climate-scenario benchmark. As noted above (see R1-5), we have removed the stronger climate framing from the title and downplayed the comparison within the text. The reviewer's point reinforces our decision to treat non-climatic drivers as important context for our findings, rather than components we can explicitly model here.
Added Text at L225-228 "Other non-climatic drivers that can significantly influence channel conveyance and flood hazard such as land-use change, sedimentation, urbanisation and dam

operations were not possible to model and isolate in the context of this study, but are still important and should be explored further in future work."

#Minor Points

• While using WorldPop to calculate the exposure there is no mention of the temporal information on the data.

We have now clarified in the manuscript that the WorldPop dataset used represents static population estimates for the reference year 2020 (Line 399). We have added a sentence that temporal population change is not included in this analysis (Line 404-406)

Temporal population change is therefore not incorporated in our exposure analysis. We also note in this revised text that modelling population dynamics is beyond the scope of this study but is an important consideration for future work.

• Figure 1: The arrows show the trend in Channel conveyance, however instead of referring to Figure 2 adding what are the different direction of the arrow means would help readers to understand better.

Changes made – thank you for the suggestions. We have added additional text in the figure caption

• LN 62: what is "c." here?

This is an abbreviation for the Latin 'circa', meaning approximately. We have removed the abbreviation and replaced it with 'approximately' to aid clarity.

• LN 224: in the Fathom model, how is the roughness represented?

We have added a reference to the methods section and revised the text (at L289-292) there as follows in order to address this point:

"Channel roughness is fixed at a Manning's coefficient of 0.035 and bathymetry is estimated given this assumption, making the model less sensitive to Manning's n than a typical flood model based on observed geometry. Floodplain roughness is fixed based on land cover as outlined by Sampson et al[7], these can vary in space but are fixed for all simulations."

Reviewer #3 (Remarks to the Author):

The study investigates a very common assumption in many GFMs that the river bankfull conveyance capacity corresponds to a 2-year return flow. For a case study of a part of the Mississippi River, the actual flow corresponding to bankfull conveyance capacity was determined as well as its temporal change. Furthermore, a sensitivity analysis was employed to compare changes in flooding from temporal changes in bankfull conveyance flow and climate change. The study found significant variation of the bankfull return flow from the often-used 2-year assumption. It also found that changes in bankfull return flow are similar to changes in projected climate change with respect to the flood hazard. This is an important study highlighting the need to better understand and incorporate river bathymetry in GFMs.

The study is well written and uses appropriate methods. However, some key assumptions are not well motivated and uncertainties throughout the experiment are not well discussed in

relation to the final conclusions as detailed below. I therefore suggest minor revision to address these points.

We thank the reviewer for these encouraging comments and their view that this is an important study. We address their requests for minor revisions below.

R3-1. In many places, you discuss channel conveyance capacity which is determined by the cross-sectional shape and roughness of the channel bed. However, in the sensitivity analysis, you seem to only include the depth. It's also not clear from the methods how the channel width and Manning roughness in the model are parametrized. Can you clarify in the manuscript what you mean by channel conveyance capacity, motivate the choice to focus on depth only, and discuss how changes in depth and roughness could affect the results?

We agree that channel conveyance capacity depends on depth, width, and roughness. In the regional variant of the Fathom GFM used here, however, these individual components are not separately parameterised. Instead, the model uses a bankfull conveyance formulation (expressed in the sensitivity analysis of Figure 4 by the Bankfull Return Period), which encapsulates width, depth, and roughness into a single lumped representation of the bankfull discharge. The definition of channel conveyance capacity is now clarified in the paper at L46-50.

R3-2. Your model is based on flows estimated from an RFFA with "6% and 29% error for the 10-year and 100-year flows". A systematic bias in the estimated return flows from the RFFA can have a significant influence on your results. Can you report the specific errors at the gauge stations in the area and if these are significant, address the effect of these biases on the results?

We thank the reviewer for raising this point. We agree that systematic biases in RFFA-derived return-period flows can influence absolute hazard estimates; however, gauge-specific error values are not available, which only reports basin-wide performance metrics (6% and 29% for the 10- and 100-year flows). We have added the following sentences from Line 302:

Return-period discharges are derived from RFFA for which only basin-scale performance metrics are available; gauge-specific uncertainties cannot therefore be quantified. While systematic bias in these estimates may affect absolute flood magnitudes, the analysis focuses on relative changes in flood hazard associated with channel-depth change, which are far less sensitive to absolute return period flow values, such that explicitly propagating return flow uncertainty would not alter the direction or relative magnitude of the reported effects.

R3-3. Uncertainties in many steps of the experiment are not accounted for or discussed in relation to the conclusions. I suggest discussing and where possible including the following uncertainties in your figures: uncertainties in the RP flows (see previous point), uncertainties in the extrapolation of the sensitivity curves (figure 4), uncertainties in the estimation of temporal changes in BFRP and the range of temporal changes in BFRP between locations, and uncertainties in changes in RP due to climate change.

We thank the reviewer for raising this important point. We agree that there are several sources of uncertainty across the workflow and have added extra details. Key uncertainties noted by the reviewer and the respected line number giving additional details about them:

- *RP Flow Uncertainty – see R3-2*

- *Temporal changes in BFRP are estimated from linear trends fitted to channel capacity time series; uncertainty arises from the fitted stage–discharge relationship and the trend estimation, which is partially conveyed through the regression statistics and confidence intervals shown in Figure 2. The range of temporal changes in BFRP between locations is illustrated directly by the differing magnitudes and directions observed across gauges. Formal quantification of between-site uncertainty would require a different modelling framework and is beyond the scope of this study.*
- *Sensitivity Curve Analysis. Line 422 we have added a new paragraph discussing the new Bayesian approach to estimate the uncertainty in the extrapolation and have added confidence intervals to the extrapolations in Figure 4 as blue shaded areas.*
- *We have added several sentences from Line 451 discussing the uncertainties of RP due to climate change:*
    - *By using the multi-model median return period for discharge values from the nine AOGCMs used in the Hirabayashi et al. study we reduce the influence of outlier AOGCM model results and minimise structure uncertainty. To estimate the return period, Hirabayashi et al used a Gumbel distribution and report good accuracy in our region with a probability plot correlation coefficient (PPCC) score of >0.95 (95% confidence interval). Exploring the full range of uncertainty from AOGCM and its propagation to flood inundation was outside the scope of this indicative level study.*

R3-4. In many places, you use the term flood risk as the population exposed for a certain return period. However, for flood risk, the impacts have to be assessed under a range of exceedance probabilities. I suggest the authors use the term flood impact instead to be more consistent with the literature.
We thank the reviewer for this helpful clarification. We agree that the term flood risk is more appropriately reserved for analyses integrating impacts across a range of exceedance probabilities. In our study, we are assessing exposure under single return-period events, which aligns more closely with the term flood impact. We have therefore updated the manuscript to instead use the term flood 'impact' (or population impacted) throughout for consistency with established terminology.

# Minor comments
L17: It is unclear what the range in underestimation of flood extents and exposed population represents. It seems related to 20 and 100-year return periods at different estimated BFRP. Can you clarify this in the text such that readers can better interpret these numbers.
We thank the reviewer for pointing this out. We agree that the meaning of the reported ranges could be clearer. We have now clarified in the manuscript that the range in underestimation of flood extents and exposed population reflects the combined results from the (added) 5, 20- and 100-year return-period simulations across the different estimated BFRP values. This explanation has been added to ensure readers can easily interpret these numbers with the added text at L17-18:
'for the 3 flood return periods analysed (5-year, 20-year and 100-year), with the greatest differences for the more frequent floods.'.

L146 (Figure 4): While care has been taken to select a model with good fit, there is (large)

statistical uncertainty in extrapolating predictions of five points. This uncertainty should be shown in the figure.

We agree that visually indicating the statistical uncertainty in the extrapolated sensitivity curve will improve clarity. We have therefore added blue shading to Figure 4 to represent the confidence band around the extrapolated curve and reported the corresponding error range at the 0.3 BFRP point in the caption and text.

Including the 5-year flood created challenges in estimating uncertainty as the approaches used estimated implausible negative flood inundation area and population exposed. Therefore, we changed our approach to the statistical fitting to give more realistic extrapolation and uncertainty estimates. Consequently, we have updated the extrapolation of flood predictors section in the Methods (L407-432) to the following text:

The lowest possible bankfull return period (BFRP) used to represent bankfull channel capacity in the Fathom GFM is 1.1 years, but estimates from stream gauges in our study region give values as low as 0.3 years. To include these low BFRP values in our analysis, we modelled the relationship between the simulated BFRP values available in the Fathom GFM (1.1, 1.5, 2, 5 and 10) and the corresponding simulated inundated areas and population exposures. Initial curve fitting using standard non-linear regression approaches and information-theoretic model selection (based on Akaike Information Criterion and residual standard error) using the 'aomisc' and 'drc' packages in R, revealed that for inundated area the Weibull 3 parameter function was the best fit and for population exposed the asymptotic and power curve parameters were the best fit. However, these well-performing functions produced implausible extrapolations when extended below the minimum modelled BFRP (0.3), including non-monotonic behaviour and exposure estimates that were inconsistent across return periods (e.g. higher exposure for the 1-in-20-year flood than for the 1-in-100-year flood). Additionally, when estimating confidence intervals, these estimates extended into implausible negative values for inundated area and population exposed.

To address these limitations, we adopted a Bayesian non-linear modelling framework with explicit structural and distributional constraints. For both inundated area and population exposure, we fitted monotonic asymptotic models using a Gamma likelihood to enforce strictly positive predictions and to better represent heteroscedasticity in the simulated outputs. Models were anchored at the lowest available modelled BFRP (1.1 years) using a high-weight pseudo-observation to ensure that extrapolations to lower BFRP values remained consistent with the simulated data. Weakly informative, data-driven priors were specified to stabilise inference given the limited number of observations available for each return period. This approach ensured monotonic, physically plausible behaviour across the full range of BFRP values, while providing coherent uncertainty estimates for extrapolated inundation and exposure metrics. All Bayesian models were fitted using the 'brms' package in R[45]. Despite our best efforts and advanced approached, our extrapolations should be treated with caution as we are extrapolating using only 5 BFRP data points.

L159: You state that the relative effects of temporal changes in conveyance and climate change on flood inundation are similar in magnitude. For this conclusion, you take the most extreme change in estimated BFRP from all gauges. Can you motivate this choice, or include a range of measured changes across all gauges in your results?

In the revised manuscript, we have now downplayed the comparison with climate-scenario changes, as suggested by referee 1 (see our response R1-5). Our response to R1-5 also details how we have revised the text to clarify our selection of the range of BFRPs used in this part of our analysis.

L267: Can you clarify why the actual observed 2011 flow was not used for validation instead of the 75-year return period flow? Since there are large spatial differences in AEP as shown in extended Data Table 1, how do you make sure you're not comparing apples and pears?

We have now clarified in the manuscript that the observed 2011 flow was not used for validation because the regional GFM operates on return-period flows rather than event-specific hydrographs, meaning the model cannot ingest the actual 2011 discharge directly. The 75-year return-period flow was selected because it is the closest operationally available layer to the magnitude of the 2011 event and therefore provides the most appropriate comparison within the model framework. We have added a clarification sentence at Line 325-326:

'The Fathom model operates on return-period flood rather than event specific hydrographs, and thus we compared to the closest return period of the Fathom model.'

Regarding spatial differences in AEP, we agree this is important to acknowledge. We now note in the text that comparisons are made within a consistent return-period framework, ensuring that we are not mixing events of different probabilistic meanings ("apples and pears"), even though local AEP estimates vary spatially in the observational record. This clarification is now included in the revised manuscript from Line 333-335:

'We acknowledge we are using a spatially varying return period event to compare against a spatially invariant return GFM simulation, thus the skill scores should be viewed as indicative of simulation quality.'

L327: You assume stationary flow return periods over the period of 1980 until present and use these to estimate the BFRP. Can you show that this is in fact an acceptable assumption for the stations assessed?

We agree that strict stationarity of flow return periods over 1980–present cannot be guaranteed. However, in this analysis stationarity of the flow distribution is not assumed to reflect physical reality, but is used deliberately as an analytical control to isolate the influence of changing channel capacity on bankfull return periods, following Slater et al. (2015). As noted in the Methods, the same flow record is used for the full period specifically to quantify how changes in channel conveyance alone alter the effective return period of bankfull flow. Any underlying non-stationarity in flows would introduce additional uncertainty but would not invalidate the interpretation of the channel-capacity effect, nor alter our central conclusion that channel evolution can materially influence flood impacts.

L335: You use changes in RPs from Hirabayashi et al. (2021) to reflect climate change in your study. However, the baseline for their study (1971-2000) is different from the baseline of your study (1980-2023). Can you discuss how this difference may affect your results and conclusions?

We thank the reviewer for highlighting this baseline difference. In the revised manuscript we now explicitly acknowledge that Hirabayashi et al. (2021) use a 1971–2000 baseline, whereas our geomorphic analysis covers 1980–2023. As part of our broader revision that reframes and downplays the climate comparison (see R1-5 and response above to the reviewer's minor comment at L159), we now present the RP changes only as an indicative benchmark in any case. Our aim is simply to illustrate that conveyance-driven changes can be of a broadly similar order of magnitude to climate-related changes reported for the same region, even if assessed

over different time periods. This clarification has been added to Line 448-451 to ensure readers interpret the comparison appropriately.

'It should be noted that the historical period of Hirabayashi et al (2021) (1971-2000) is different to our geomorphic analysis (1980-2023), and thus the absolute changes in flood impacts that we report should be viewed more as indicative rather than absolute results.'

L339: I suggest renaming this section to "extrapolation of flood predictors" which is more descriptive of the content as you also fit different curves in other sections of the methodology.

We thank the reviewer for this helpful suggestion. We agree that "extrapolation of flood predictors" is a more accurate and descriptive title for this section, given that multiple curve-fitting steps occur elsewhere in the methodology. We have updated the section heading accordingly in the revised manuscript.

L363: You use the multi-model median, but how did you deal with the spatial distribution of changes in return period flow as shown in Extended Data Figure 2-3.

Thank you for highlighting the lack of clarity around this point. We represented the spatial distribution of changes in return period by extracting the return flood map for each cell (as shown in Extended Data Figure 2-3) in the climate change simulations. In other words, if the return period in a cell changed from a 20–year period flood to a 50 year flood, we extracted the 50-year flood map from the Fathom flood maps. Then we stitched all these maps together to obtain a spatially distributed climate change impacted flood map.

We have added further information in the Methods section at L445-448: These inundation maps for the end of the century (2071-2100) were created by extracting the closest return period Fathom flood map for each cell in the Hirabayashi et al (2021) data, and stitching these maps together to form a climate change impacted flood map that has spatially distributed changes in return periods.

Reproducibility: Some manual data filtering was done for the streamflow transect data (L301) and no scripts seem to be provided to reproduce the analysis. It would be strongly recommended to share the (filtered) pre-processed data if possible as well as the code to make the research reproducible.

We thank the reviewer for this suggestion and agree that transparency around data handling is important. The manual filtering applied to the streamflow transect data was extremely limited and used only to remove a very small number of clearly non-physical measurements that lie far outside the range of the observed data and are visually obvious in the transect plots. For example, at site 07022000 (third row of Figure 2), a small number of stage values exceeding 50 m were excluded; these values are orders of magnitude outside the observed stage–discharge relationship and have no plausible physical interpretation. Given how far removed these points are from the data cloud, we do not consider it informative to enumerate all such thresholds explicitly. These minimal quality-control steps do not affect the interpretation of the results.

Reviewer responses

REVIEWERS' COMMENTS:

Reviewer #1 (Remarks to the Author):

I think that the authors addressed very well the points that I raised in my first review of this manuscript (as well as the points/suggestions of the other two reviewers). Overall, I am happy with the new version of the paper. The aims and novelties of the paper are clear and the outcomes are of interest for a large audience.
Nicola Surian

Many thanks for spending the time to review our article and providing helpful and insightful comments. It has really helped improve the article.

Reviewer #3 (Remarks to the Author):

The authors have substantially strengthened the manuscript in this revision. In particular, the improved discussion of uncertainties in the approach enhances the robustness and transparency of the analysis. The authors have also done a good job of better positioning their findings. At this stage, only a few minor comments remain that, once addressed, will further refine the clarity and presentation.

L15. I suggest clarifying that your findings are based on seven gauges in a case study of the Mississippi floodplain.

Thank you for your suggestion. We have needed to reduce the abstract size, but have just about included your suggestion.

L152. The limitation of the Fathom model in representing bankfull return periods (BFRP) below 1.1 years due to its reliance on annual maxima for the RFFA is clear. However, for this experiment the model could have been based on peaks-over-threshold (POT) extreme values from local gauges to overcome this limitation and work around the constraints of the global Fathom model. Please provide reasoning for why you decided to use the RFFA and extrapolate the flood predictors from only five data points instead. In addition, if the actual BFRP is often found to be smaller than 1.1 years, please add a discussion on how to incorporate this in large-scale flood models using RFFA based on annual maxima going forward.

Thank you for your suggestion. We have included the following sentence at Line 175.

Using the peaks-over-threshold (POT) approach to calculate extreme values could theoretically be used to calculate bankfull return periods below 1.1 years and thus negate the need to extrapolate results below 1.1 years, but it was out of the scope of this study to re-calculate bankfull return periods using this method, especially as the annual maxima approach is widely used in GFMs.

River Channel Change Can Affect Flood Hazard and ~~Risk as Much as Climate~~Impact Substantially

Laurence Hawker, Stephen E. Darby, Louise Slater, Daniel R. Parsons, Richard J. Boothroyd, Philip J. Ashworth, Hannah Cloke, Pauline Delorme, Solomon H. Gebrechorkos, Helen Griffith, Yukiko Hirabayashi, Julian Leyland, Yinxue Liu, Stuart J. McLellland, Jeffrey Neal, Andrew P. Nicholas, Greg Sambrook Smith, Chris Sampson, Ellie Vahidi, Michel Wortmann, and Dai Yamazaki

Over a billion people are at risk of flooding[1,2], and this ~~will~~is expected to double by 2050 due to climate change, population growth, and encroachment into at-risk areas[3,4]. Global Flood Models (GFMs) are vital tools for producing flood hazard maps[5,6,7] supporting key policy interventions. However, GFMs often assume that river bankfull flow capacity corresponds to a fixed return period (RP), of typically two years, which overlooks spatial and temporal variations in channel size, shape, and roughness.

To quantify the extent to which channel variability biases GFM predictions we employ a ~~typical~~ GFM, ~~use~~ the Fathom model[7,8], in a sensitivity analysis that evaluates how inundated areas and population exposures respond when forced with empirically-derived bankfull capacities. We find that actual RPs of present-day bankfull flows (<1 year) differ ~~significantly~~substantially from the assumed two-year RP, leading to underestimations of flood extent (9–~~59~~152%) and exposed populations (15–~~118%).~~472%) for the 3 flood return periods analysed (5-year, 20-year and 100-year), with the greatest differences for the more frequent floods. We also show that, over multi-decadal timescales, changes in past channel morphology ~~rival or exceed~~are comparable to the influence of future climate change ~~on~~in affecting flood ~~risk~~impacts, depending on emissions scenarios. ~~The spatial-temporal evolution of bankfull capacity is therefore a critical control on flood hazard and risk, making it vital that GFMs represent river channel variability and change accurately.~~

Flooding has devastating impacts on people and the environment. During 2002-2021, flood events globally impacted >80 million people and caused average annual financial losses exceeding US$41 billion[9]. Moreover, flood hazard is projected to grow in the future, driven in part by the increasing frequency of extreme precipitation events under human-induced warming[10]. ~~Flood risk, the product of flood hazard and the vulnerability and exposure~~The number of people and value of assets~~,~~ exposed to flooding is also projected to increase substantially due to demographic changes, economic growth and encroachment of people into flood-prone areas[3]. For example, it has been estimated that by 2050 over two billion people will live in areas where the annual probability of flooding exceeds 1%[2]. Within this context, the accurate prediction of flood hazard and ~~risk~~impacts is essential to enable effective policy interventions such as the development of early warning systems[11,12] and insurance evaluations[13,14].

Flood models have significantly improved over the past decade, with enhancements in skill being driven by improved digital elevation datasets[15,16], better estimates of regional extreme events[17], advances in computation[18], and more sophisticated process representation[7,19,20,21]. However, our ability to model future changes in flood hazard and risk remains poor. ~~A critical limitation is that existing global-scale flood models (GFMs)~~Local-scale modelling incorporates detailed representations of relevant hydraulic processes and provide high fidelity information but their spatial coverage is limited, rendering their application to regional or national scale analyses challenging. Global-scale flood models (GFMs) offer a way to fill that coverage gap and have been employed in applications ranging from national-scale climate risk assessments[22], humanitarian response planning[23,24] and catastrophe risk evaluations[21]. However, these larger-scale models are frequently challenged by data scarcity and a critical limitation is that existing GFMs represent river morphology in unrealistic ways. For example, GFMs are initialised by representing channel morphological properties, such as depth and roughness, by assuming that the bankfull flow-carrying capacity of the river channel can be equated to a discharge with a specified return period (RP) that is spatially and ~~temporally[22]~~temporally[25] invariant, and which is typically assumed to take a value of 2-years[7]. This bankfull discharge is sometimes referred to (as in this paper) as the channel conveyance capacity. As such, GFMs view river channels essentially as 'static pipes', in which channel conveyance varies neither through space nor time. Yet, as discussed further below, observations show that this assumption of spatial invariance is incorrect (Figure 1) and many rivers are also undergoing morphological ~~changes[23]~~changes[26] that are

substantial enough to affect channel conveyance capacity and thus potentially alter flood ~~hazard[24,25,26].~~hazard[27,28,29]. However, the extent to which spatial and temporal variations in channel conveyance capacity propagate through to biased GFM predictions of flood hazard and ~~risk remains unknown~~impacts remains unknown. Specifically, the direction and scale of the impacts arising cannot readily be estimated, so it is important to quantify the emergent behaviour arising from including variations in channel conveyance capacity within GFMs.

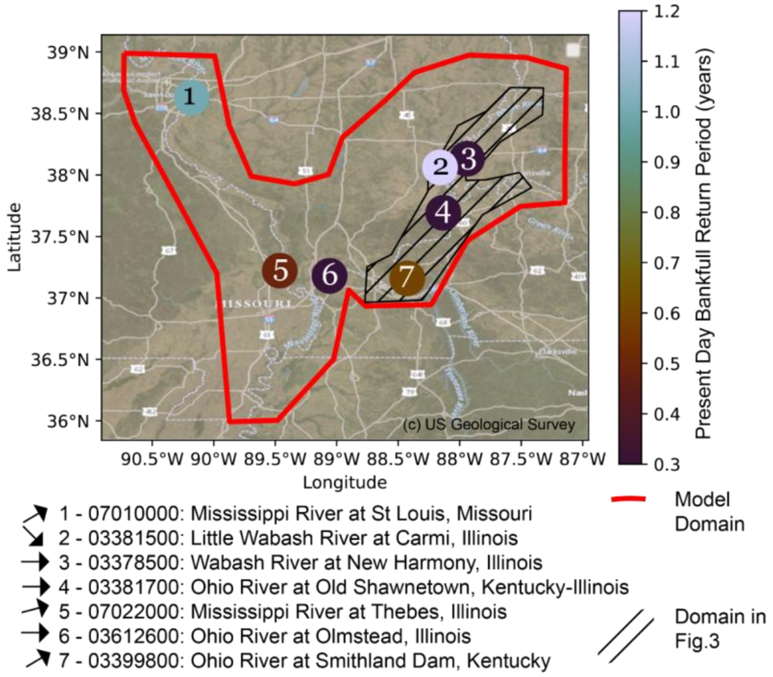

Figure 1: *Map showing the extent of the model domain (red boundary line) used to simulate part of the 2011 Mississippi River flood event, showing locations of stream gauges employed in the study. Empirically estimated Bankfull ~~flow return periods~~Flow Return Periods (BFRPs; indicated by the colours of the symbols) and trends in channel conveyance capacity (indicated by directions of the arrows, with an arrow facing up showing an increase in bankfull channel capacity, horizontal no/little change and down a decrease in channel conveyance capacity) during the period of record are shown for each gauge (detailed analyses in Figure 2 and Extended Data Figure 1). The hashed area shows the area of the model domain highlighted in Figure 3.*

To address this question, here we quantify how: (i) different model-imposed bankfull conveyance capacities, relative to the commonly assumed 2-year RP, and; (ii) multi-decadal changes in bankfull conveyance, driven by river channel evolution, affect GFM predictions of flood hazard and ~~risk. We also compare GFM flood hazard predictions as driven by river channel evolution to those forced by future climate change, showing that the two effects have similar magnitudes.~~impacts. To do this we employ the Fathom Global Flood Model[7,8] (see Methods) to a 135,000 km^2 region of the Mississippi River floodplain, where approximately 10 million people and US$3.4 billion of assets are at risk of flooding. ~~This area~~The Fathom model is similar to other GFMs in its representation of channel conveyance (Methods), but the version of the model employed here is a regional scale model developed for use specifically across the United States. We focus on the Mississippi floodplain as a case study because this region has experienced many floods, most notably during spring ~~2011[27]~~2011[30], an event which had an estimated return period of ~~c.~~approximately 80~~-~~ years (Extended Data Table 1). We evaluated the performance of the Fathom ~~GFM~~model simulations for the 2011 flood by

comparing simulated inundation extents against those estimated from remotely sensed imagery (within the validation region delimited by the hashed area in Figure 1) (see Methods). This comparison yielded Critical Success Index (CSI; see Methods) scores ranging between 0.584 and 0.602 depending on the imposed bankfull conveyance capacity (Extended Data Table 2). These are relatively high CSI scores (see Methods), lending confidence in the Fathom ~~GFM's~~model's ability to simulate flooding within the study area accurately. We then used the validated Fathom ~~GFM~~model in a sensitivity analysis that varied imposed bankfull return periods (BFRPs) while holding all other variables constant. This sensitivity analysis was repeated for ~~two~~three different driving flow discharges, chosen to represent ~~a~~relatively frequent (5-year return period flow) moderate (20-year return period) and ~~a~~ much larger (100-year) ~~flood~~floods, respectively.

The range of BFRPs (1.1 to 10 years) employed in our sensitivity analyses was selected to include values above and below the typically assumed value of 2-years. We also determined the actual range of empirically-derived present-day BFRPs within our study region, using United States Geological Survey (USGS) stream gauge data (Figure 1). Specifically, for each gauge we estimated (see Methods): (i) bankfull discharge flows; (ii) their present-day return periods, and; (iii) the evolution of these return periods through time. The present-day BFRP values so-obtained are *not* spatially-invariant (Figure 1), with values ranging from 0.3 to 1.2 years, a substantial deviation from ~~oft~~the often-cited 2-year value. Four of the seven gauges within the model domain also exhibit statistically significant ($p<0.05$) *temporal* trends in BFRP (three examples are shown in Figure 2; see Extended Data Figure 1 for all gauges). We return to the significance of these trends below.

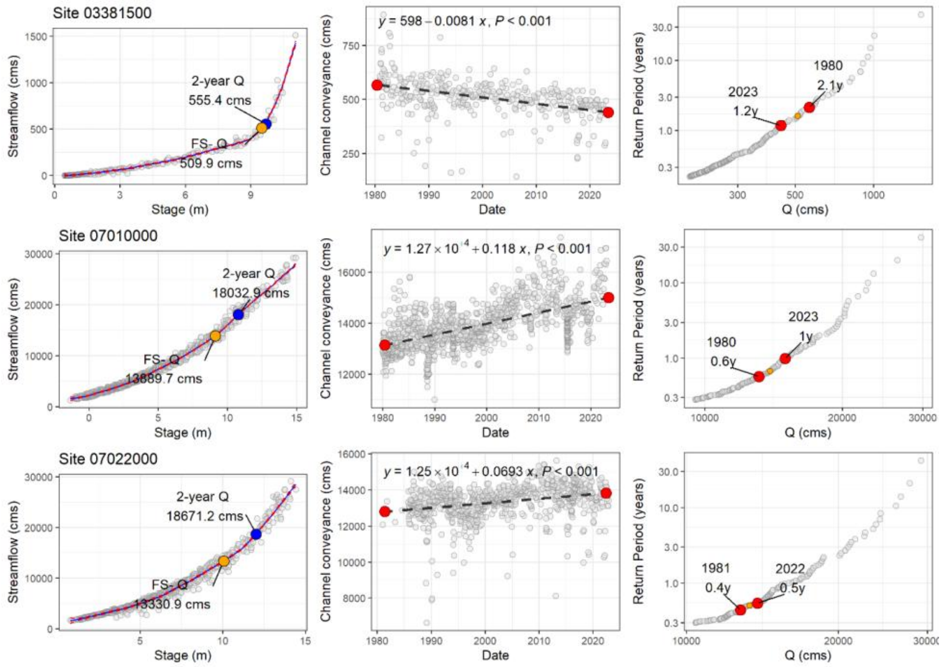

Figure 2: *Examples of three gauges (rows) in the study region with decreasing (03381500) and increasing (07010000 and 07022000; rows) significant (p<0.05) trends in bankfull channel capacity over time.* ***Left column*** *shows the transect measurements of stage and streamflow with a fitted Loess curve (blue line) and confidence intervals (red dashed lines), along with the estimated streamflow value at flood stage ("bankfull channel capacity", orange circle)* ~~*and the*~~*with an empirically estimated two-year exceedance flow (blue circle).* ***Middle column*** *shows channel capacity values over time with the first and last values (red circles) extracted from the fitted linear regression (blue line).* ***Right column*** *shows estimated bankfull return periods at the start and end of the record.*

To provide an initial illustration of the substantial impact of the choice of model-imposed bankfull conveyance on flood hazard, Figure 3 maps flood depths and extents simulated by the Fathom ~~GFM~~model for BFRPs of 1.1 and 5.0 years under ~~each of~~ the 20- and 100-year forcing flows. For both these flood magnitudes the simulated inundation is ~~more~~greater when the smaller ~~bankfull return period~~BFRP flow is used in the ~~GFM~~model, due to the out-of-bank flows being reached sooner (simulated flood extents are annotated on each panel of the figure). Note that the difference in flood extents simulated across the two different BFRPs plotted here is greater for the moderate (20-year) than the extreme (100-year) flood (4,803 and 2,767 km^2 respectively; see Figure 3a,b versus Figure 3d,e). That is, the model flood inundation extent sensitivity to changes in river channel capacity is greater for smaller floods. This reflects the diminishing proportion of the total flow conveyed within the river channels, as opposed to over the floodplain, as flood magnitude increases. For reference, Figure 3 also highlights the flood inundation extents simulated for a future (2071-2100) climate change scenario (Shared Socio-economic Pathway 2 / Representative Concentration Pathway 4.5; see Methods) and using an imposed bankfull return period of 2~~-~~ years. We use these climate change projections later to compare their impacts in driving Fathom GFM simulated flood extents to those forced by changing channel conveyance.

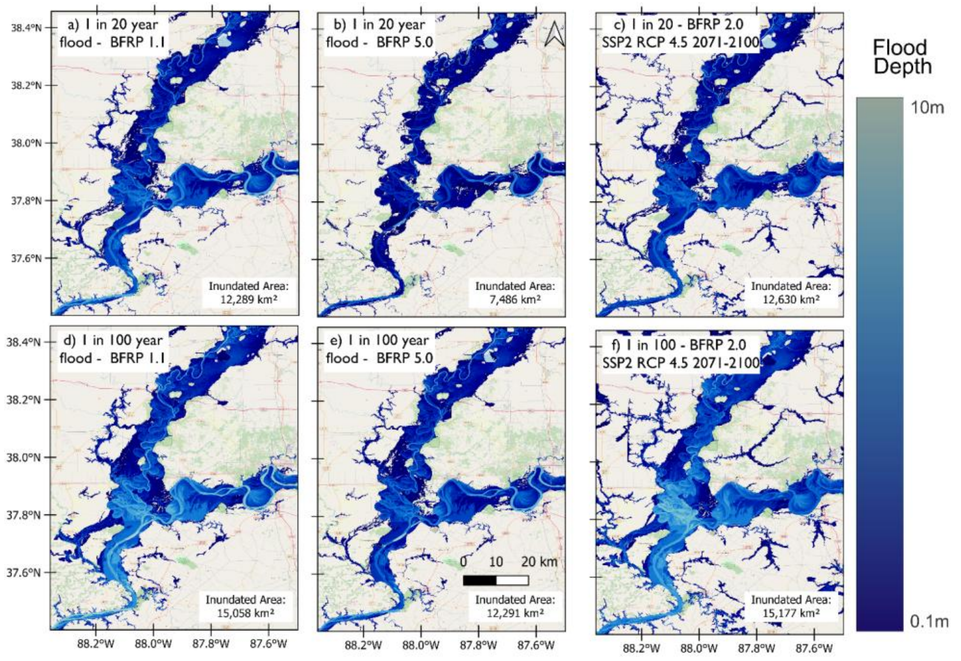

Figure 3: *Inundation maps under varying climate forcing and channel conveyance capacities as represented by bankfull return periods (BFRP) of 1.1 (panels a and b) and 5.0 (panels d and e) years, for discharges corresponding to the 20 (panels a-c) and 100-year (panels d-f) floods for the hashed area shown in Figure 1. Panels c & f correspond to flooding in 2071-2100 for the SSP 2 RCP 4.5 climate change scenario from ref. 6, and with an imposed BFRP of 2-years. Note that the inundated areas annotated on each panel refer to results for the whole model domain (the red area in Fig. 1) and not just for the zoomed in sections shown. Dominant flow direction is north to south.*

Figure 4 presents the full results of our sensitivity analysis, which we use initially to demonstrate the large potential ~~GFM~~model biases arising from the choice of BFRP used to represent the river channel morphology. This sensitivity analysis shows how the Fathom ~~GFM flood hazard (Figure 4,b) and risk (Figure 4c,d)~~flood predictions vary in response to channel conveyance capacity, as indexed here using the ~~bankfull return period (~~BFRP~~)~~ flow. Note that we also estimate flood ~~risk based on~~impact in terms of the populations exposed to

flooding, as determined using WorldPop ~~data[28]~~data[31], in the simulated inundated areas (see Methods). On each diagram the darker grey vertical bands highlight the range of present-day conveyance capacities as estimated at the stream gauges in our area of interest (see Figure 2 and Extended Data Figure 1). This range of ~~actual~~empirically-derived bankfull return periods of 0.3 to 1.1 years differs substantially from the 2-year value commonly employed in GFMs. These differences, when combined with the sensitive response of the Fathom ~~GFM~~model to changes in the imposed BFRP, allow the biases in flood hazard and ~~risk~~impact estimates arising from the choice of BFRP to be determined. Since the model sensitivity is dependent on the magnitude of the forcing flood (Figure 3; discussed above), these computed biases are also flood-magnitude dependent, being greater for smaller floods. For example, the inundated areas simulated for the 2-year BFRP, denoted by the ~~orange~~yellow symbols on Figure 4, are 6,220 km^2, 10,204 km^2 and 13,781 km^2 for the 5-year, 20-year and 100-year floods, respectively. In contrast, when imposing the more-realistic range of observed present-day BFRP values of 1.1 to 0.3-years, simulated inundation extents increase to between 9,306 and 15,654 km^2 (a 50 to 151% increase compared to the 2-year BFRP), 12,289 and ~~16,176~~14,506 km^2 (a 20 to ~~59~~42% increase compared to the 2-year BFRP) and to between 15,058 and ~~17,308~~16,697 km^2 (a 9% to ~~26~~21% increase) for the 5-year, 20-year and 100-year floods, respectively (Figure 4a,b,c; Extended Data Table 3). In terms of flood ~~risk~~impacts, the computed biases are even greater. Specifically, for the 20-year flood the number of people exposed increases from 119,059 for the baseline 2-year BFRP scenario to between 181,283 and ~~259,613~~278,244 (a 52 to ~~118~~133.7% increase across the range of present-day observed BFRPs), while for the 100-year flood the number of people exposed grows from 205,817 for the baseline 2-year BFRP scenario to between 237,162 and ~~316,856~~273,296 (a 15 to ~~54~~33% increase) (Figure ~~4c,d~~4e,f; Extended Data Table 3). For the more frequently recurring 5-year flood, the computed biases of people exposed as a percentage are even greater at between 104,588 and 197,865 (a 149 to 472% increase). Note that the upper end of the bias ranges quoted here, which correspond to the 0.3-year BFRP, are uncertain. This is because, like other ~~GFMs~~large-scale flood models, the Fathom ~~GFM~~model used here is unable to represent bankfull return periods below 1.1 years (see Methods), so our simulations are thus truncated at this threshold. We therefore estimated the flood inundation extent and populations exposed for BFRPs below 1.1 years via extrapolation (dashed lines, with confidence intervals shaded in blue on Figure 4) along best-fit curves to the model results (see Methods).

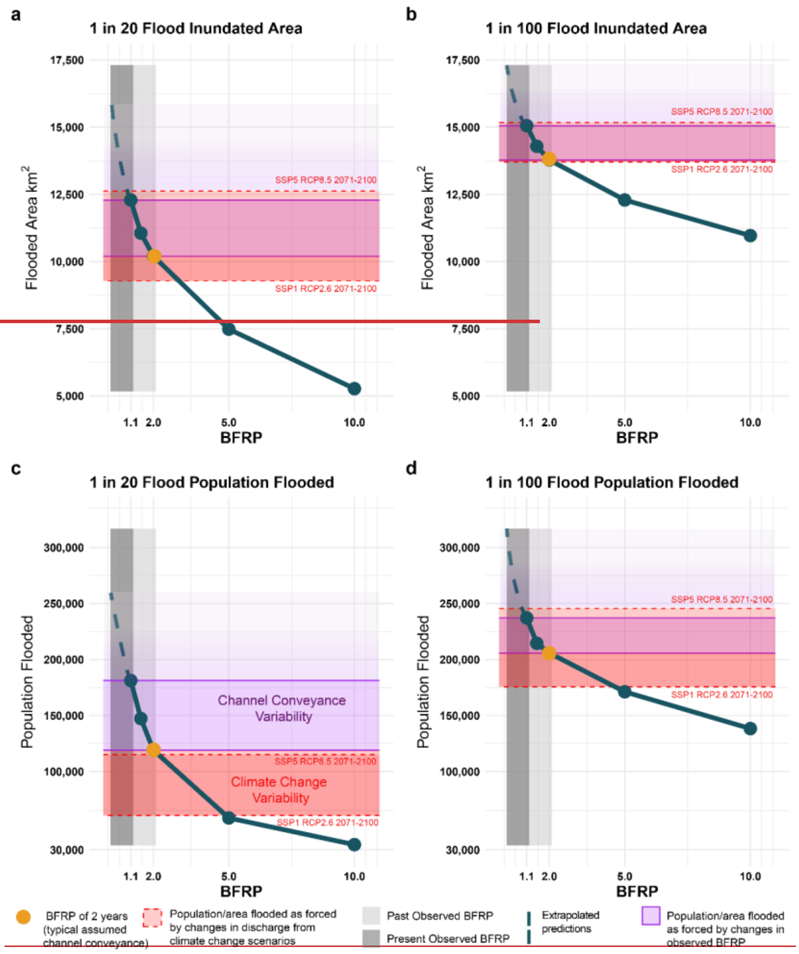
a
1 in 20 Flood Inundated Area
Flooded Area km^2
SSP5 RCP8.5 2071-2100
SSP1 RCP2.6 2071-2100
BFRP
b
1 in 100 Flood Inundated Area
Flooded Area km^2
SSP5 RCP8.5 2071-2100
SSP1 RCP2.6 2071-2100
BFRP
c
1 in 20 Flood Population Flooded
Population Flooded
Channel Conveyance Variability
SSP5 RCP8.5 2071-2100
Climate Change Variability
SSP1 RCP2.6 2071-2100
BFRP
d
1 in 100 Flood Population Flooded
Population Flooded
SSP5 RCP8.5 2071-2100
SSP1 RCP2.6 2071-2100
BFRP
BFRP of 2 years (typical assumed channel conveyance)
Population/area flooded as forced by changes in discharge from climate change scenarios
Past Observed BFRP
Present Observed BFRP
Extrapolated predictions
Population/area flooded as forced by changes in observed BFRP

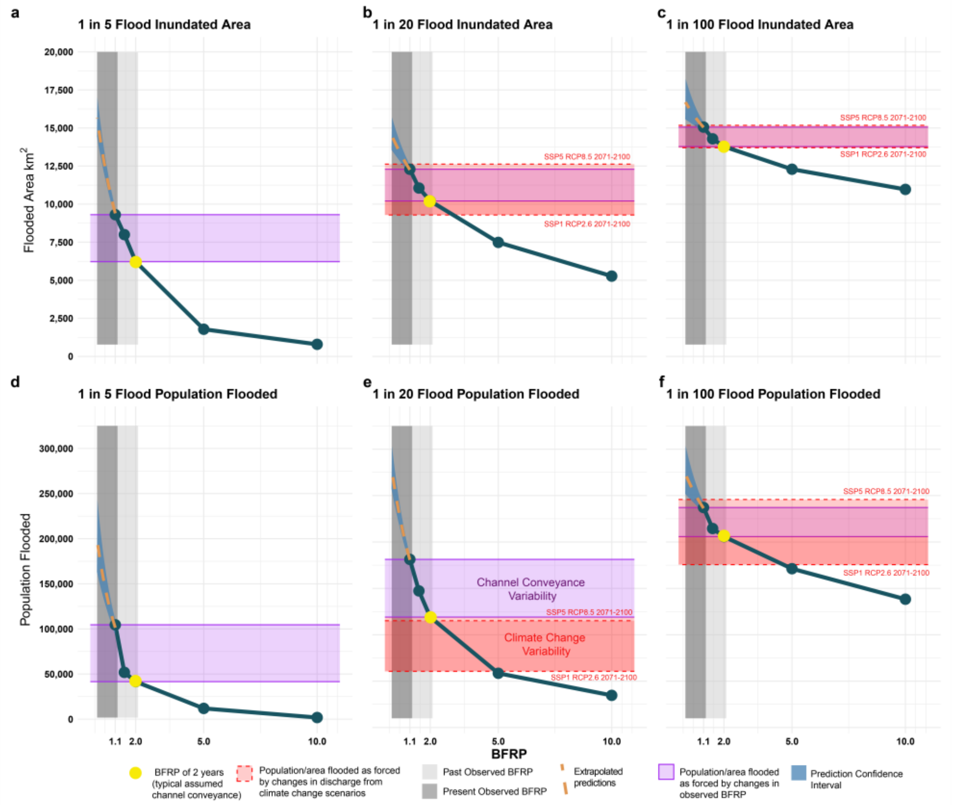

Figure 4: *Sensitivity of simulated inundated area (top row) and population exposed to flooding (bottom row) to changes in channel conveyance capacity for ~~20~~5-year (left column), 20-year (middle column) and 100-year (right column) floods (blue lines). Note that the dashed ~~portions of the blue~~orange lines for bankfull return periods (BFRPs) less than 1.1 years are based on curve-fits to the modelled data, with the blue shaded area corresponding to computed confidence intervals (see Methods). ~~Note that the~~The dark grey vertical band represents the range of present-day bankfull conveyance capacities (BFRPs) estimated at the seven stream gauges in the study area (0.3 to 1.2 years; see right hand column of Extended Data Figure 1) and is compared to the 2-year bankfull return period flow (shown as ~~an orange~~a yellow symbol). The range of values within the red shading indicates simulated inundated areas/populations exposed under moderate (SSP1 RCP2.6; lower bound) and intense (SSP5 RCP8.5; upper bounds) future (2071-2100) climate change scenarios and for a bankfull channel conveyance capacity of 2~~-years.~~ years (note that these climate change scenarios are only available for 20-year and 100-year floods). Purple shaded areas indicate simulated inundated areas/populations exposed when bankfull channel conveyance is varied according to the historical trends in BFRP observed at the stream gauges in the study area as indicated here by the light grey vertical bands~~)~~.*

Finally, we undertake an indicative comparison of the relative effects of *temporal* changes in river channel conveyance capacity ~~on our estimates of~~versus future climate change on flood inundation and population exposure~~ are similar to those forced by future climate change~~. To ~~show~~do this we first quantified the influence of future climate by adjusting the return periods of the two (20- and 100-year) present-day flows driving the Fathom ~~GFM~~model for the period 2071-2100[6], based on the Coupled Model Intercomparison Project Phase 6 (CMIP6) climate simulations (see Methods). For our study area, our three future climate scenarios show moderate ~~variation~~variations in return period, with the SSP1 RCP2.6 scenario showing a

decrease in return period (Extended Data Figures 2 and 3). The change in inundated area and people exposed between the present day and future (2071-2100) climates, when imposing a 2-year RP channel conveyance capacity, is then indicated by the red shaded areas on Figure 4 (with numeric values listed in Extended Data Table 3). To quantify the ~~impact~~possible impacts of~~ past~~ river channel evolution on flood extent and exposed populations, ~~and based on~~we imposed BFRPs of 2.1 and 1.2 years within the model. While we do not claim that this imposed reduction in channel conveyance is representative of the actual historical evolution of the river channel network in our model domain over the last four decades, we argue that the magnitude of the imposed change is plausible, and perhaps even conservative. This is because the selected range of imposed BFRPs is consistent with historical data ~~from our ~~between the years 1980 and 2023 as observed at the stream gauges ~~(~~in our study area (see top right panel of Figure 2~~), we used bankfull return periods of 2.1 and 1.2 years for the years 1980 and 2023, respectively. These~~). Yet, stream gauges are often sited at locations believed to be relatively stable and so morphological changes elsewhere could even be larger. Nevertheless, we frame this part of our analysis as an indicative comparison only.

The past changes in channel conveyance, represented by the light grey shading on Figure 4, force changes in inundated area and population exposure as indicated by the purple shading on the diagrams (with numeric values listed in Extended Data Tables 4, 5 and ~~5~~6 for the 5-, 20- and 100-year forcing flows, respectively). A comparison of the red versus purple shaded areas thus indicates the potential relative effects of ~6 decades of potential future climate change versus ~4 decades of past channel evolution on inundation extent and populations exposed. For the moderate (20-year RP) driving flow discharge, the declining bankfull capacity results in a substantial predicted historical increase in flood extent of ~~421~~403 km^2 per decade that is comparable, or greater, in magnitude to the changes forced by future climate change, which range from -150 km^2 (SSP1 RCP2.6) to +404 km^2 per decade (SSP5 RCP8.5) across the forcing climate scenarios (Extended Data Table ~~4~~5). Note that the ~~increase~~increases in flood extent due to the ~~past~~imposed reductions in channel capacity potentially offset the declines in flood extent simulated for the two more moderate future climate scenarios, but compound the increase projected for the higher intensity (SSP5 RCP8.5) climate change scenario. This pattern of response is evident also for the more extreme (100-year RP) driving flow discharge, albeit with more muted rates of change: +~~237~~262 km^2 per decade under the morphological forcing and -13 to +233 km^2 per decade (depending on scenario) under future climate change (Extended Data Table ~~5~~6). Changes in flood ~~risk~~impact forced by past morphological versus future climate changes are also therefore similar in magnitude. For example, for the moderate (20-year RP) the number of people exposed to flooding increases by 12,~~411~~523 per decade due to the ~~historical evolution of~~imposed reductions in bankfull capacity but declines between 9,741 and 641 per decade (depending on scenario) under future climate change (Extended Data Table ~~4~~5). For the more extreme (100-year RP) flood, the increase in flood ~~risk~~impact due to the ~~historical~~imposed channel evolution (+~~5,747~~6,077 people per decade) is likewise comparable to the change (-5,037 to +6,664 people per decade, depending on scenario) forced by future climate change (Extended Data Table ~~5).~~6). Inundation extent and population exposed were also calculated for the relatively frequent flood (5-year RP) (Extended Table 4) but could not be compared to climate change simulations as the climate forcing was not available for this return period. Other non-climatic drivers that can significantly influence channel conveyance and flood hazard such as land-use change, sedimentation, urbanisation and dam operations were not possible to model and isolate in the context of this study, but are still important and should be explored further in future work.

Overall, our study shows that channel conveyance capacity variability matters in estimating flood hazard and ~~risk~~impacts. We find that neither of the common assumptions that GFMs employed to represent channel conveyance capacity ~~by ~~(using a bankfull discharge with a return period that is spatially invariant and which has an assumed value of 2~~-~~ years) is supported by empirical evidence. By running our Fathom ~~GFM~~model simulations across the range of present-day bankfull return periods actually observed in our study area, we obtain substantial bias in simulated flood hazard, with inundated areas estimated to be between 50 and 152% greater for the relatively frequent 5-year flood, between 20 and ~~59~~42% greater for a moderate 20-year flood, and between 9 and ~~26~~21% greater for a more extreme 100-year flood, when using more realistic bankfull return periods informed by gauge data. Biases in flood ~~risk~~impacts are even greater, with the number of people estimated to be exposed to flooding between: (i) 149 to 472% greater for a relatively frequent 5-year flood; (ii) 52 and 118% greater for a moderate 20-year flood, and~~ between~~; (iii) 15 and 54% greater for

the 100-year flood. ~~Our second key~~An ancillary finding is that the effect of historical evolution of channel morphology on flood hazard and ~~risk~~impacts is potentially greater in our case study area than the effect of future climate-driven changes under a low (SSP1 RCP2.6) and a moderate (SSP2 RCP4.6) emissions scenario, for both moderate (20-yr RP) and extreme (100-yr RP) floods. Even under a high emissions scenario (SSP5 RCP8.5), the evolution of flood hazard and ~~risk~~impacts forced by future climate change is approximately equal in magnitude to the evolution of flood extent and exposure forced by ~~past~~imposed changes in bankfull capacity that are comparable to those observed in the study region in the last four decades.

While we acknowledge that our study focuses on a single study area and employs a specific, regional-scale variant of a GFM, we argue that the implications of our findings are broadly applicable. This is because the Fathom ~~GFM~~model estimates channel conveyance in a similar way to other GFMs[19,~~29,30~~32,33] , implying that their model sensitivities are likely similar to those quantified here. Moreover, recent work has shown that many other rivers, not just those in the Mississippi region, have bankfull flow return ~~period flows~~periods that deviate substantially from 2-years. Specifically, Liu et al.[~~31~~34] have shown that the majority (64.6%) of 8,519 gauging station locations worldwide have bankfull flows with return periods that are either less than 1-year or greater than 3-years. Moreover, other work (Slater et al., 2015)[27] has shown that temporal changes in bankfull return periods occur across multiple stream gauge locations in the United States, not just at the specific locations employed in our study. This means that the existing approach of GFMs utilising a temporally and spatially invariant 2-year flow return period ~~flow~~ to represent bankfull conveyance capacity is incorrect and likely to lead to substantial biases in estimates of flood hazard across many of the world's floodplains. ~~As such, we urgently need new and improved representations of the size and shapes of river channels, and their variations through time and space, within GFMs~~As such, we now need to employ emergent datasets such as Liu et al.[34] to provide new and improved representations of the conveyance capacity of river channels. In addition, water-surface profiles from the Surface Water and Ocean Topography (SWOT) satellite[35,36] offer great potential to enhance model calibration and bathymetry inversion. However, these opportunities essentially offer a better way to characterise a static snapshot of the river morphology and therefore there is an urgent need to generate time-varying estimates of conveyance capacity globally within GFMs in order to complement these advances and expand the scope of the 'proof of concept' investigation that we present here to the global river network.

Methods

Flood Model Simulations

Flood inundation was simulated using the Fathom Global Flood Model (GFM)[7,8] at 1 arc second (~30 m) grid spacing. The model is underpinned by LISFLOOD-FP, which solves the local inertial formulation of the shallow water equations[20,~~32~~37]. The version of the model developed in this study is the same as ref.8. Elevation data is predominantly taken from the USGS National Elevation Dataset (NED) at 1 arc second grid spacing (~30 m), augmented with LiDAR terrain data where available. River channels are modelled in one dimension, which allows for river channels of any size to be represented (even below model grid spacing). The location of rivers is delineated by a flow accumulation grid created from the elevation data and USGS National Hydrography Dataset. Even in this relatively well measured region, river bathymetry is largely unknown and we thus parametrize channel conveyance by assuming the channel can convey a certain return period discharge. Normally, the channel conveyance is assumed to be the 1 in 2-year return period flow, but herein we undertake a sensitivity analysis in which we alter the channel capacity to convey 5 different return period flows: 1.1, 1.5, 2, 5 and 10 years, respectively. Bed elevation is then estimated using an inverted gradually varying ~~solver[33]~~solver[38] with ~~bankful~~bankfull elevation extracted using a 3x3 moving widow from land pixels from the DEM adjacent to the water mask. Channel roughness is fixed at a Manning's coefficient of 0.035 and bathymetry is estimated given this assumption, making the model less sensitive to Manning's n than a typical flood model based on observed geometry. Floodplain roughness is fixed based on land cover as outlined by Sampson et al[7], these can vary in space but are fixed for all simulations. Lastly, return period discharges, used for both channel bed estimation and flow input for flood simulations were determined using a regional flood frequency analysis (RFFA). We used the methods and data set of Bates et al. ~~(2021; ref. 34),~~[39] , which took the annual maxima discharge from 6,902 USGS river gauges and grouped them by proximity and

hydrologically similarity to calculate index flow for every pixel in the flow accumulation array. Note that, by using the annual maxima discharge, we could not calculate flow return periods below 1 year. Consequently the bankfull channel capacity used in the ~~GFM~~flood model could not match the values estimated empirically from the gauges (see *Estimation of Bankfull Return Periods,* below). This is an inherent limitation of the RFFA used to drive the ~~GFM~~model. Extreme flows are estimated by deriving growth curves from the pooled gauges, with a 6% and 29% error for the 10-year and 100-year flows, ~~respectively~~[34]~~.~~respectively[39]. However, this error is dampened by coupling the channel conveyance estimate and RFFA, as the channel conveyance will account for an over or underestimate of extreme flow by the RFFA (i.e., if the RFFA is underestimated then the channel conveyance will be lower). ~~We simulated 2 return period flows: the~~Return-period discharges are derived from RFFA for which only basin-scale performance metrics are available; gauge-specific uncertainties cannot therefore be quantified. While systematic bias in these estimates may affect absolute flood magnitudes, the analysis focuses on relative changes in flood hazard associated with channel-depth change, which are far less sensitive to absolute return period flow values, such that explicitly propagating return flow uncertainty would not alter the direction or relative magnitude of the reported effects. We simulated 3 return period flows: the 1 in 5, 1 in 20 and 1 in 100-year return period flows for the 5 channel conveyance capacities (i.e., channels able to convey the 1 in 1.1; 1.5; 2; 5; and 10-year flows). These ~~two~~three driving return period flows were chosen as they represent a relatively frequent flood (1 in 5-years), a moderate flood (1 in 20-years) and the flood defence return period flow (1 in 100-years).

Model Validation

To test the quality of the model, we compared simulations of the 2011 flood event to inundation extents observed by remote sensing. Google Earth Engine was used to estimate flood inundation extents from Landsat 5 Thematic Mapper imagery acquired during the 2011 flood. Partially cloud-free imagery was available for sections of the Mississippi River at Wabash-Ohio on 3rd May 2011 and Cairo-Tiptonville on 10th May 2011. The CFmask algorithm was applied to mask obstructions from cloud and cloud shadow ~~pixels~~[35]pixels[40] and the modified normalized difference water index (~~MNDWI~~[36]MNDWI[41]) was used to indicate the presence of water. Based on manual testing, a MNDWI threshold of 0 was defined to discriminate between water and non-water ~~pixels~~[37]pixels[42]. To ensure that water pixels were associated with floodwaters from the main channels, only water pixels connected to centerlines from the Global River Widths from Landsat (GRWL) ~~Database~~[38]Database[43] were included in the Landsat-derived flood footprints used for model validation purposes.

The model simulations used in the validation were driven by a flow discharge corresponding to the 1 in 75-year return period flow to match, as closely as possible, the estimated return period of the 2011 event. The Fathom model operates on return-period flood rather than event specific hydrographs, and thus we compared to the closest return period of the Fathom model. In fact, calculated Annual Exceedance Probabilities (AEPs) exceed 0.2 within some of the tributary channels, but with an average of 2 to 4 within the Mississippi mainstem (see Extended Data Table 1). Nevertheless, an estimated AEP within the test domain, based on data from 5 gauges, results in an estimated mean AEP of ~1.2 (95% Confidence Intervals of 0.75 and 3.85), and thus a representative flood reoccurrence interval of ~80 years for the ~~event~~[39]event[44] (Extended Data Table 1). The 1 in 75-year flood return period was used in the simulation as this return period is the closest available within the suite of existing GFM simulations employed in this study[8]. We acknowledge we are using a spatially varying return period event to compare against a spatially invariant return model simulation, thus the skill scores should only be viewed as indicative of simulation quality. Within the validation itself, we employed the widely used Critical Success Index (CSI) ~~metric~~[40]metric[45] to assess the skill of the simulations (see Extended Data Table 2), as the CSI accounts for both under and over-prediction, with a score of 0 indicating no skill and a score of 1 indicating a perfect model. However, CSI scores above 0.7 are rarely ~~obtained~~[41]obtained[46]. For regional scale models like the one used in this study, CSI scores of 0.56-0.67[7], 0.78[34] and 0.65-0.76[21] have been obtained in prior studies. Unlike local scale models, these regional-scale (and indeed global-scale models) have not been extensively calibrated to local conditions. Other metrics reported in Extended Data Table 2 were computed following Wing et al. (2017~~; ref. 42).~~) [47]. These metrics include the Hit Rate (HR, ranges from 0 indicating no skill to 1 indicating god skill), which tests the proportion of the observed wet areas replicated by the model and thus measures the model's tendency to underpredict flood hazard; the Miss Rate (defined as 1 – HR), and; the False Alarm Ratio (FAR, ranges from 0 indicating no

false alarms to 1 indicating all false alarms), which indicates the proportion of the observed wet areas that are not inundated in the model and thus measures the model's tendency to overpredict flood extent.

Estimation of Bankfull Return Periods and their Change Through Time

Streamflow transect data were downloaded for all gauges within the model domain from the US Geological Survey National Water Information System (NWIS), and bankfull stage values were obtained for each of the sites from the National Water Prediction Service of the National Oceanic and Atmospheric Administration (NWS, NOAA). Of the available gauges, one (03322420; the Ohio River at Uniontown Dam, Kentucky) was discarded from the analysis due to the lack of recent data, leaving a total of seven gauges employed in the analysis. Of these seven sites, one gauge (03381500; the Little Wabash River at Carmi, Illinois), did not have a NWS estimate of flood stage, so a value of 9.5 metres was employed based on visual breakpoint analysis of the stage-discharge and stage-width transect data at that site. For all the seven sites, the stage-discharge and stage-width relationships were checked visually to ensure that the flood stage corresponded to a breakpoint, with discharge or width increasing visibly above the flood stage value. However, the presence of a breakpoint in the data was more visible at some sites than others, and it is possible in some sites that the NWS moderate flood stage value is a closer approximation of bankfull. All data were trimmed to the period 1980-01-01 to present, and a small handful of extreme~~ (visibly erroneous) measurements were removed~~, clearly non-physical measurements were removed (e.g., isolated stage or width values lying far outside the observed stage-discharge relationship), as these were visually obvious artefacts and did not represent plausible channel geometry.

Channel capacity analyses were then undertaken following the methods of Slater et al. (2015)~~[24]~~[27], summarised here briefly. First, a Locally weighted scatterplot smoothing curve (Loess) was fit to the stage-discharge transect data and used to extract bankfull discharge at flood stage based on the NWS flood stage value (see Figure 2, left column). Next, channel capacity at flood stage was estimated at each point in time (see Figure 2 middle column) by adding the residuals of the stage-discharge relationship to the estimated bankfull discharge. This approach assumes the channel conveyance capacity is homoscedastic and behaving similarly (e.g., increasing) across different values of stage. Channel capacity values were then plotted over time, and a linear regression fitted to assess which sites exhibit significant trends in channel capacity. Finally, channel conveyance capacity values were extracted for the first and last years of the data from the linear regression and assumed to represent average channel capacity at the start and end dates (e.g., in 1980 and 2022) of the analysis.

Flow return periods were computed by downloading the daily streamflow data from the USGS NWIS for the period 1980-01-01 to present. To compute flow return periods we retained only years with at least 95% complete data (i.e. more than 346 values). We used a common threshold of the 80th percentile of all daily data at each site as the baseline threshold from which to extract flood peaks (we note that the final estimated return periods are not sensitive to the choice of threshold; results using the 70th percentile differed by only 3%). Flood peaks were then extracted following the approach recommended by the Water Resource Council of the USA, as described in Lang et al. (1999~~; ref. 43),~~)[48], where the flood threshold separation is computed as 4 days + the logarithm of the drainage area of each site in square kilometres, with a minimal intermediate flow of less than 75% of the lowest of the two peaks. Return periods were computed by ranking the flood peaks in descending order with the 'average' ties method and computing (n + 1)/rank, where n represents the total number of identified flood peaks. A Loess curve was employed to fit the empirical relationship between the peak flow values and the estimated return periods. Finally, the return periods were extracted for the bankfull channel capacity values in the start year and end year from this empirical relationship. Note that this approach estimates the influence of changing the channel capacity relative to a stationary flow distribution; i.e., the same flow record is used for the full time ~~period[24].~~period to isolate the effect of changing channel capacity[27].

Estimation of Population Exposure

We estimated the number of people living (based on 100 m spatial resolution building constrained WorldPop ~~data[28]~~data for the year 2020[31]) within the flood extents simulated by the various conveyance and driving flow return period combinations. WorldPop data was downscaled to 1 arc second (to match the flood model

resolution) and overlayed with binary flood maps. To create the binary flood maps, we used a threshold flood depth of 15cm, and thus water depth above 15cm was considered flooded. To calculate population exposure, we used a raster calculator to multiple the downscaled population with the binary flood maps. This workflow was implemented in R. Temporal population change is not incorporated in our exposure analysis as modelling population dynamics is beyond the scope of this work, but can be an important driver of future flood exposure[49].

Curve-Fitting
Extrapolation of Flood Predictors

The lowest possible bankfull return period (BFRP) used to represent bankfull channel capacity in the Fathom GFM is 1.1 years, but empirically-derived estimates from stream gauges in our study region give values as low as 0.3 years. To include these low BFRP values in our analysis, we fitted non-linear regression models linking the modelled the relationship between the simulated BFRP values (available in the range Fathom model (1.1 to 10 years), 1.5, 2, 5 and 10) and the Fathom GFMcorresponding simulated inundated areas and associated population exposures. Various functions were tested and assessed using the Initial curve fitting using standard non-linear regression approaches and information-theoretic model selection (based on Akaike Information Criterion (AIC) and residual standard error measures. The best function selected) using the 'aomisc' and 'drc' packages in R, revealed that for inundated area was the Weibull 3 parameter function. For flood exposure, was the best fit and for population exposed the asymptotic and power curve parameters were the best fit. However, these well-performing functions produced some evaluated functions, including the Weibull 3, that gave optimal fits (as assessed using the AIC and residual standard error metrics) deliveredinstances of implausible predictions whereby the estimated exposures extrapolations when extended below the minimum modelled BFRP (0.3), including non-monotonic behaviour and exposure estimates that were unrealisticallyinconsistent across return periods (e.g. higher exposure for the 1-in-20-year flood than for the 1-in-100-year flood. As such, we selected the two best functions that generated more plausible outcomes, namely the asymptotic function for the 1 in 20-year flood and the power curve function). Additionally, when estimating confidence intervals, these estimates extended into implausible negative values for the 1 in 100-year flood. In all cases curve fitting was carried out using the 'aomisc' and 'drc' packages in Rinundated area and population exposed.

To address these limitations, we adopted a Bayesian non-linear modelling framework with explicit structural and distributional constraints. For both inundated area and population exposure, we fitted monotonic asymptotic models using a Gamma likelihood to enforce strictly positive predictions and to better represent heteroscedasticity in the simulated outputs. Models were anchored at the lowest available modelled BFRP (1.1 years) using a high-weight pseudo-observation to ensure that extrapolations to lower BFRP values remained consistent with the simulated data. Weakly informative, data-driven priors were specified to stabilise inference given the limited number of observations available for each return period. This approach ensured monotonic, physically plausible behaviour across the full range of BFRP values, while providing coherent uncertainty estimates for extrapolated inundation and exposure metrics. All Bayesian models were fitted using the 'brms' package in R[50]. Despite our best efforts and advanced approached, our extrapolations should be treated with caution as we are extrapolating using only 5 BFRP data points.

Future Climate Change Projections

We quantified the potential influence of typical future climate change projections on our model simulations by using the change in return period estimates from Hirabayashi et al. (2021)[6]. These altered return period estimates for the period 2071-2100 are based on the Coupled Model Intercomparison Project Phase 6 (CMIP6) climate simulations. Hirabayashi et al. (ref. 2021)[6]) estimated return periods for the past (1971–2000) and future (2071-2100) by extracting daily runoff data from multiple atmosphere–ocean general circulation models (AOGCM) from CMIP6, routing the runoff through a global river network using CaMa-Flood v4.0[44]0[51] and fitting a two parameter Gumbel extreme value distribution. Three climate scenarios were used in the Hirabayashi et al. (ref. 2021)[6]) study (SSP1-RCP2.6, SSP2-RCP4.5, SSP5-RCP8.5) and were subsequently used in this analysis. Flood inundation projections for the end of the century (2071-2100) were then created by estimating the multi-model median return period for discharge values corresponding to both the 1 in 20 and 1 in 100-year floods over the historical period (1971-2000). These inundation maps for the

end of the century (2071-2100) were created by extracting the closest return period Fathom flood map for each cell in the Hirabayashi et al (2021) data, and stitching these maps together to form a climate change impacted flood map that has spatially distributed changes in return periods. It should be noted that the historical period of Hirabayashi et al (2021) (1971-2000) is different to our geomorphic analysis (1980-2023), and thus the absolute changes in flood impacts that we report should be viewed as indicative rather than absolute results. By using the multi-model median return period for discharge values from the nine AOGCMs used in the Hirabayashi et al. study we reduce the influence of outlier AOGCM model results and minimise structure uncertainty. To estimate the return period, Hirabayashi et al used a Gumbel distribution and report good accuracy in our region with a probability plot correlation coefficient (PPCC) score of >0.95 (95% confidence interval). Exploring the full range of uncertainty from AOGCM and its propagation to flood inundation was outside the scope of this indicative level study.

Data Availability

Fathom global flood model data are available for academic purposes and were provided by Fathom. The WorldPop constrained high-resolution population counts are available to download online (https://hub.worldpop.org/doi/10.5258/SOTON/WP00685). Google Earth Engine (GEE) codes to extract flood footprints from Landsat imagery for model validation are available here (https://code.earthengine.google.com/30e8f4a66cf99b815c135b87d1429946 and https://code.earthengine.google.com/09ca689dfb8e5df65f48cad5121b43c4). Gauging station data was obtained from the USGS National Water Information System (NWIS; https://waterdata.usgs.gov/nwis), with the channel capacity data retrieved using the dataretrieval R package[45]. package[52].

References

1. Di Baldassarre, G., Kooy, M., Kemerink, J.S. and Brandimarte, L., 2013. Towards understanding the dynamic behaviour of floodplains as human-water systems. Hydrol. Earth Syst. Sci., 17, 3235-3244. DOI: 10.5194/hess-17-3235-2013
2. Jongman, B., Wagemaker, J., Romero, B.R. and Coughlan de Perez, E. 2015. Early Flood Detection for Rapid Humanitarian Response: Harnessing Near Real-Time Satellite and Twitter Signals. ISPRS Int. J. Geo-Inf., 4(4), 2246-2266. DOI: 10.3390/ijgi4042246
3. Rentschler, J., Salhab, M. & Jafino, B.A., 2015. Flood exposure and poverty in 188 countries. Nature Communications, 13, 3527. DOI: 10.1038/s41467-022-30727-4
4. IPCC, 2023. Climate Change 2023: Synthesis Report. Contribution of Working Groups I, II and III to the Sixth Assessment Report of the Intergovernmental Panel on Climate Change [Core Writing Team, H. Lee and J. Romero (eds.)]. IPCC, Geneva, Switzerland, 184 pp., DOI: 10.59327/IPCC/AR6-9789291691647.
5. Alfieri, L., Bisselink, B., Dottori, F., Naumann, G., de Roo, A., Salamon, P., Wyser, K. and Feyen, L. 2017. Global projections of river flood risk in a warmer world. Earth's Future, 5, 171-182. DOI: 10.1002/2016EF000485
6. Hirabayashi, Y., Tanoue, M., Sasaki, O. et al. 2021. Global exposure to flooding from the new CMIP6 climate model projections. Scientific Reports, 11, 3740. DOI: 10.1038/s41598-021-83279-w
7. Sampson, C.C., Smith, A.M., Bates, P.D., Neal, J.C., Alfieri, L. and Freer, J.E., 2015. A high-resolution global flood hazard model. Water resources research, 51(9), 7358-7381. DOI: 10.1002/2015WR016954
8. Wing, O.E., Lehman, W., Bates, P.D., Sampson, C.C., Quinn, N., Smith, A.M., Neal, J.C., Porter, J.R. and Kousky, C., 2022. Inequitable patterns of US flood risk in the Anthropocene. Nature Climate Change, 12(2), 156-162. DOI: 10.1038/s41558-021-01265-6
9. Centre for Research on the Epidemiology of Disasters (CRED). 2023. 2022 Disasters in numbers, CRED, Brussels. Available at: https://cred.be/sites/default/files/2022_EMDAT_report.pdf
10. Intergovernmental Panel on Climate Change (IPCC). 2023. *Climate Change 2023: Synthesis Report. Contribution of Working Groups I, II and III to the Sixth Assessment Report of the Intergovernmental Panel on Climate Change* [Core Writing Team, H. Lee and J. Romero (eds.)]. IPCC, Geneva, Switzerland, 184 pp. DOI: 10.59327/IPCC/AR6-9789291691647.
11. Emerton, R., Cloke, H., Ficchi, A., Hawker, L., de Wit, S., Speight, L., Prudhomme, C., Rundell, P., West, R., Neal, J. and Cuna, J., 2020. Emergency flood bulletins for Cyclones Idai and Kenneth: A critical evaluation of the use of global flood forecasts for international humanitarian preparedness and response. International Journal of Disaster Risk Reduction, 50, 101811. DOI: 10.1016/j.ijdrr.2020.101811
12. World Meteorological Organization (WMO), 2022. Early Warnings for All: The UN Global Early Warning Initiative for the Implementation of Climate Adaptation. Executive Action Plan 2023-2027. WMO, Geneva.
https://library.wmo.int/idurl/4/58209
13. Franco, G., Becker, J.F. and Arguimbau, N. 2020. Evaluation methods of flood risk models in the (re)insurance industry. Water Security, 11, 100069. DOI: 10.1016/j.wasec.2020.100069.
14. Boudreault, M., Grenier, P., Pigeon, M., Potvin, J.M. and Turcotte, R., 2020. Pricing flood insurance with a hierarchical physics-based model. North American Actuarial Journal, 24(2), 251-274. DOI: 10.1080/10920277.2019.1667830
15. Yamazaki, D., Ikeshima, D., Tawatari, R., Yamaguchi, T., O'Loughlin, F., Neal, J.C., Sampson, C.C., Kanae, S. and Bates, P.D., 2017. A high-accuracy map of global terrain elevations. Geophysical Research Letters, 44(11), 5844-5853. DOI: 10.1002/2017GL072874
16. Hawker, L., Uhe, P., Paulo, L., Sosa, J., Savage, J., Sampson, C. and Neal, J., 2022. A 30 m global map of elevation with forests and buildings removed. Environmental Research Letters, 17(2), 024016. DOI:10.1088/1748-9326/ac4d4f
17. Zhao, G., Bates, P., Neal, J. and Pang, B., 2021. Design flood estimation for global river networks based on machine learning models. Hydrology and Earth System Sciences, 25(11), 5981-5999. DOI: 10.5194/hess-25-5981-2021
18. Shaw, J., Kesserwani, G., Neal, J., Bates, P. and Sharifian, M.K., 2021. LISFLOOD-FP 8.0: the new discontinuous Galerkin shallow-water solver for multi-core CPUs and GPUs. Geoscientific Model Development, 14(6), 3577-3602. DOI: 10.5194/gmd-14-3577-2021

19. Yamazaki, D., Kanae, S., Kim, H. and Oki, T., 2011. A physically-based description of floodplain inundation dynamics in a global river routing model. Water Resources Research, 47(4), W04501. DOI: 10.1029/2010WR009726
20. Neal, J., Schumann, G. and Bates, P., 2012. A subgrid channel model for simulating river hydraulics and floodplain inundation over large and data sparse areas. Water Resources Research, 48(11). DOI: 10.1029/2012WR012514
21. Bates, P.D., Savage, J., Wing, O., Quinn, N., Sampson, C., Neal, J. and Smith, A., 2023. A climate-conditioned catastrophe risk model for UK flooding. Natural Hazards and Earth System Sciences, 23(2), 891-908. DOI: 10.5194/nhess-23-891-2023
22. Wing, O.E., Bates, P.D., Quinn, N.D., Savage, J.T., Uhe, P.F., Cooper, A., Collings, T.P., Addor, N., Lord, N.S., Hatchard, S. and Hoch, J.M., 2024. A 30 m global flood inundation model for any climate scenario. Water Resources Research, 60(8), p.e2023WR036460. DOI:10.1029/2023WR036460
23. Rentschler, J., Salhab, M. and Jafino, B.A., 2022. Flood exposure and poverty in 188 countries. Nature communications, 13(1), p.3527. DOI:10.1038/s41467-022-30727-4
24. Emerton, R., Cloke, H., Ficchi, A., Hawker, L., de Wit, S., Speight, L., Prudhomme, C., Rundell, P., West, R., Neal, J. and Cuna, J., 2020. Emergency flood bulletins for Cyclones Idai and Kenneth: A critical evaluation of the use of global flood forecasts for international humanitarian preparedness and response. International Journal of Disaster Risk Reduction, 50, p.101811. DOI: 10.1016/j.ijdrr.2020.101811
~~22.~~25. Schumann, G., Stampoulis, D., Smith, A.M., Sampson, C.C., Andreadis, K.M., Neal, J.C. and Bates, P.D. 2016. Rethinking flood hazard at the global scale. Geophysical Research Letters, 43, 249-256. DOI: 10.1002/2016GL070260
~~23.~~26. Wu, Q., Ke, L., Wang, J. et al. 2023. Satellites reveal hotspots of global river extent change. Nat Commun **14**, 1587. https://doi.org/10.1038/s41467-023-37061-3
~~24.~~27. Slater, L. J., Singer, M.B. and Kirchner, J.W., 2015. Hydrologic versus geomorphic drivers of trends in flood hazard, Geophys. Res. Lett., 42, 1–7. DOI:10.1002/2014GL062482
~~25.~~28. Li, Y., Wright, D. B., & Byrne, P. K. (2020). The influence of tropical cyclones on the evolution of river conveyance capacity in Puerto Rico. Water Resources Research, 56(9), e2020WR027971.
~~26.~~29. Ahrendt, S., Horner-Devine, A. R., Collins, B. D., Morgan, J. A., & Istanbulluoglu, E. 2022. Channel conveyance variability can influence flood risk as much as streamflow variability in western Washington state. Water Resources Research, 58(6), e2021WR031890.
~~27.~~30. Camillo, C.A. 2012. Divine Providence: The 2011 Flood in the Mississippi River and Tributaries Project. Mississippi River Commission.
~~28.~~31. Bondarenko M., Kerr D., Sorichetta A., and Tatem, A.J., 2020. Census/projection-disaggregated gridded population datasets, adjusted to match the corresponding UNPD 2020 estimates, for 183 countries in 2020 using Built-Settlement Growth Model (BSGM) outputs. WorldPop, University of Southampton, UK. ~~doi~~DOI:10.5258/SOTON/WP00685
~~29.~~32. Ward, P.J., Jongman, B., Weiland, F.S., Bouwman, A., van Beek, R., Bierkens, M.F., Ligtvoet, W. and Winsemius, H.C. 2013. Assessing flood risk at the global scale: model setup, results, and sensitivity. *Environmental research letters*, *8*(4), p.044019. DOI: 10.1088/1748-9326/8/4/044019
~~30.~~33. Dottori, F., Salamon, P., Bianchi, A., Alfieri, L., Hirpa, F.A. and Feyen, L. 2016. Development and evaluation of a framework for global flood hazard mapping. *Advances in Water Resources*, *94*, 87-102. DOI: 10.1016/j.advwatres.2016.05.002
~~31.~~34. Liu, Y., Wortmann, M., Hawker, L. et al. 2024. Global estimation of river bankfull discharge reveals distinct flood recurrences across different climate zones. PREPRINT (Version 1) available at Research Square ~~[https://doi.org/10.21203/rs.3.rs-5185659/v1]~~[https://doi.org/10.21203/rs.3.rs-5185659/v1]
35. Fu, L.L., Pavelsky, T., Cretaux, J.F., Morrow, R., Farrar, J.T., Vaze, P., Sengenes, P., Vinogradova-Shiffer, N., Sylvestre-Baron, A., Picot, N. and Dibarboure, G., 2024. The surface water and ocean topography mission: A breakthrough in radar remote sensing of the ocean and land surface water. Geophysical Research Letters, 51(4), p.e2023GL107652. DOI: 10.1029/2023GL107652
36. Andreadis, K.M., Coss, S.P., Durand, M., Gleason, C.J., Simmons, T.T., Tebaldi, N., Bjerklie, D.M., Brinkerhoff, C., Dudley, R.W., Gejadze, I. and Larnier, K., 2025. A first look at river discharge estimation from SWOT satellite observations. Geophysical Research Letters, 52(9), p.e2024GL114185. DOI:10.1029/2024GL114185

~~32.~~37. Bates, P.D., Horritt, M.S. and Fewtrell, T.J., 2010. A simple inertial formulation of the shallow water equations for efficient two-dimensional flood inundation modelling. Journal of Hydrology, 387(1-2), 33-45. DOI: 10.1016/j.jhydrol.2010.03.027

~~33.~~38. Neal, J., Hawker, L., Savage, J., Durand, M., Bates, P. and Sampson, C., 2021. Estimating river channel bathymetry in large scale flood inundation models. Water Resources Research, 57(5), e2020WR028301. DOI: 10.1029/2012WR012514

~~34.~~39. Bates, P.D., Quinn, N., Sampson, C., Smith, A., Wing, O., Sosa, J., Savage, J., Olcese, G., Neal, J., Schumann, G. and Giustarini, L., 2021. Combined modeling of US fluvial, pluvial, and coastal flood hazard under current and future climates. Water Resources Research, 57(2), e2020WR028673. DOI: 10.1029/2020WR028673

~~35.~~40. Foga, S., Scaramuzza, P.L., Guo, S., Zhu, Z., Dilley, R. D., Beckmann, T., Schmidt, G.L., Dwyer, J.L., Huges, MJ and Brady, L., 2017. Cloud Detection Algorithm Comparison and Validation for Operational Landsat Data Products. Remote Sensing Environ. 194, 379-390. DOI:10.1016/j.rse.2017.03.026

~~36.~~41. Xu, H., 2006. Modification of Normalised Difference Water Index (NDWI) to Enhance Open Water Features in Remotely Sensed Imagery. Int. J. Remote Sensing, 27 (14), 3025-3033. DOI:10.1080/01431160600589179

~~37.~~42. Ji, L., Zhang, L., and Wylie, B., 2009. Analysis of dynamic thresholds for the normalized difference water index. Photogrammetric Engineering & Remote Sensing, 75(11), 1307-1317. DOI: 10.14358/PERS.75.11.1307

~~38.~~43. Allen, G.H., and Pavelsky, T.M., 2018. Global extent of rivers and streams. Science, 361(6402), 585-588. DOI:10.1126/science.aat0636

~~39.~~44. Driscoll, D.G., Southard, R.E., Koening, T.A., Bender, D.A. and Holmes, R.R., 2014. Annual Exceedance Probabilities and Trends for Peak Streamflows and Annual Runoff Volumes for the Central United States During the 2011 Floods. Chapter D of 2011 Floods of the Central United States. Professional Paper 1798–D, US Geological Survey.

~~40.~~45. Schaefer, J.T., 1990. The critical success index as an indicator of warning skill. Weather Forecast, 5, 570–575.

~~41.~~46. Schumann, G., Bates, P.D., Horritt, M.S., Matgen, P. and Pappenberger, F., 2009. Progress in integration of remote sensing–derived flood extent and stage data and hydraulic models. Reviews of Geophysics, 47(4). DOI: 10.1029/2008RG000274

~~42.~~47. Wing, O.E.J., Bates, P.D., Sampson, C.C., Smith, A.M., Johnson, K.A. and Erickson, T.A., 2017. Validation of a 30 m resolution flood hazard model of the conterminous United States. Water Resources Research, 53, 7968-7986. DOI: 10.1002/2017WR020917

~~43.~~48. Lang, M., Ouarda, T.B.M.J. and Bobée, B., 1999. Towards operational guidelines for over-threshold modelling. Journal of Hydrology, 225(3-4), 103-117. DOI: 10.1016/S0022-1694(99)00167-5

49. Rogers, J.S., Maneta, M.P., Sain, S.R., Madaus, L.E., & Hacker, J.P. 2025 The role of climate and population change in global flood exposure and vulnerability. Nature Communications, 16, 1287. DOI: 10.1038/s41467-025-56654-8

50. Bürkner P. C. 2017. brms: An R Package for Bayesian Multilevel Models using Stan. Journal of Statistical Software. 80(1), DOI:10.18637/jss.v080.i01

~~44.~~51. Yamazaki, D., Rendel, M., Zhou, X. and Tomoko Nitta, T., 2021. global-hydrodynamics/CaMa-Flood_v4: CaMa-Flood (v4.00). Zenodo. https://doi.org/10.5281/zenodo.4609655

~~45.~~52. Hirsch, R. M., and De Cicco, L. A., 2015. User guide to Exploration and Graphics for RivEr Trends (EGRET) and dataRetrieval: R packages for hydrologic data (No. 4-A10). US Geological Survey.

Acknowledgements

This work is part of the Evolution of Global Flood Hazard and Risk (EVOFLOOD) project supported by the Natural Environment Research Council through awards to DP (NE/S015795/2), SED and JL (NE/S015817/1), LS (NE/S015728/1), JN (NE/S015639/1), GSS (NE/S015736/1), APN (NE/S015612/1), HC (NE/S015590/1), SM (NE/S015795/2) and PJA (NE/S015655/1).

Authors and Affiliations

1. **School of Geographical Sciences, University of Bristol, Bristol, BS8 1SS, UK**

Laurence Hawker & Jeffrey Neal

2. **School of Geography and Environmental Science, University of Southampton, Southampton, SO17 1BJ, UK**
   Stephen E. Darby & Julian Leyland
3. **Geography and Environment, Loughborough University, Loughborough, UK**
   Daniel R. Parsons
4. **School of Geography and the Environment, University of Oxford, Oxford, UK**
   Solomon Gebrechorkos, Yinxue Liu, Louise Slater & Michel Wortmann
5. **School of Applied Sciences, University of Brighton, Sussex, BN2 4AT, Brighton, UK**
   Philip J. Ashworth
6. **Department of Geography, Faculty of Environment, Science and Economy, University of Exeter, Exeter, EX4 4RJ, UK**
   Andrew Nicholas & Ellie Vahidi
7. **School of Geography, Earth and Environmental Sciences, University of Birmingham, Birmingham, B15 2TT, UK**
   Greg Sambrook Smith
8. **Department of Geography and Planning, School of Environmental Sciences, University of Liverpool, Liverpool, L69 7ZT, UK**
   Richard J. Boothroyd
9. **Geography and Environmental Science, University of Reading, Reading, UK**
   Hannah Cloke & Helen Griffith
10. **Energy and Environment Institute, University of Hull, Hull, UK**
    Pauline Delorme & Stuart McLelland
11. **Fathom, Clifton Heights, Clifton, Bristol, UK, BS8 1EJ**
    Chris Sampson
12. **Institute of Industrial Science, The University of Tokyo, 4-6-1 Komaba, Meguro-ku, Tokyo, 153-8505, Japan**
    Dai Yamazaki
13. **Deparment of Civil Engineering, Shibaura Institute of Technology, Tokyo, Japan 3-7-5, Toyosu, Koto-ku, Tokyo 135-8548**
    Yukiko Hirabayashi

Author Contributions

DP, SED, LS and LH conceived the study. LH undertook the model simulations and conducted the formal analyses, with input from SED, DP, JN, CS and LS. LS undertook the analysis of bankfull conveyance capacities, while RB undertook the satellite image analyses used in model validation. DY and YH provided the climate change data. LH, SED, DP and LS led on drafting the initial manuscript and all authors contributed to its further development (writing and editing).

Extended Data

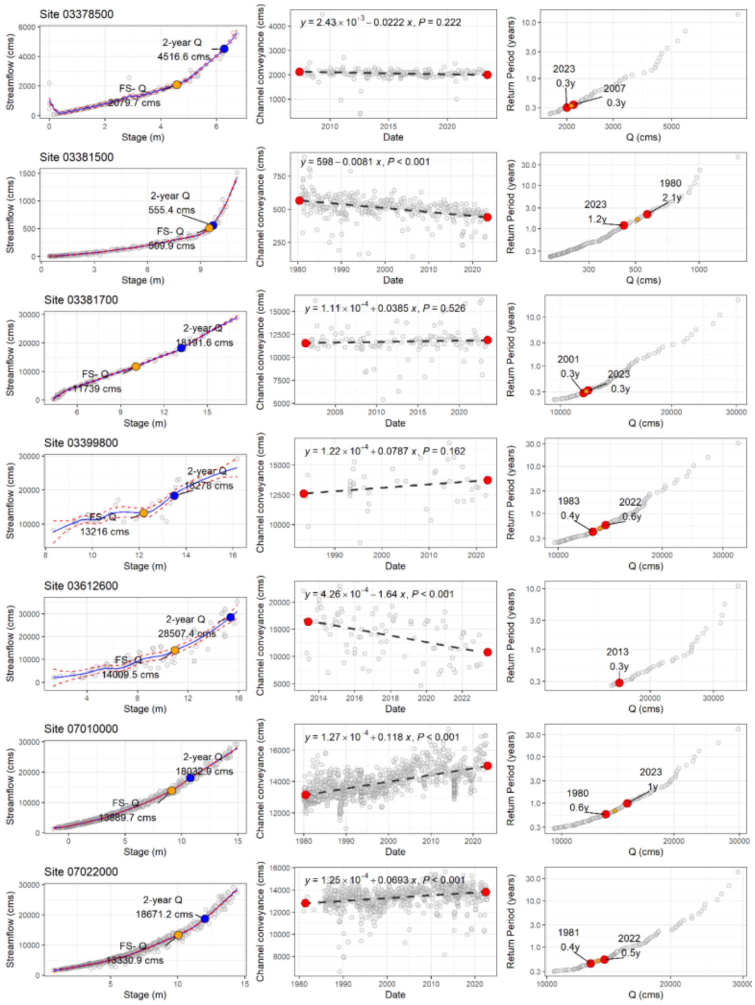

Extended Data Figure 1: *Influence of changes in bankfull channel conveyance capacity on bankfull flow return period. Rows show seven gauges (03378500: Wabash River at New Harmony, Illinois; 03381500: Little Wabash River at Carmi, Illinois; 03381700: Ohio River at Old Shawnetown, Kentucky-Illinois; 03399800: Ohio River at Smithland Dam, Kentucky; 03612600: Ohio River at Olmstead, Illinois; 07010000: Mississippi River at St Louis, Missouri, and 07022000: Mississippi River at Thebes, Illinois). Left column shows the transect measurements of stage and streamflow with a fitted Loess curve (blue line) and confidence intervals (red dashed lines), along with the estimated streamflow value at flood stage ("bankfull channel capacity", orange circle) ~~and the~~with an empirically estimated two-year exceedance flow (blue circle). Middle column shows channel capacity values over time, estimated by adding the residuals of the stage-discharge curve to the estimated bankfull discharge, following Slater et al. (2015) (ref. 24). The first and last values of channel capacity (red circles) are extracted from the fitted linear regression (blue line). Regression statistics are indicated at the top of the panel. Right column shows the empirically estimated return periods ~~of~~associated with channel capacity at the start and end of the record, with associated dates. Grey circles indicate flood peaks extracted over the period of record and their corresponding return period (see Methods).*

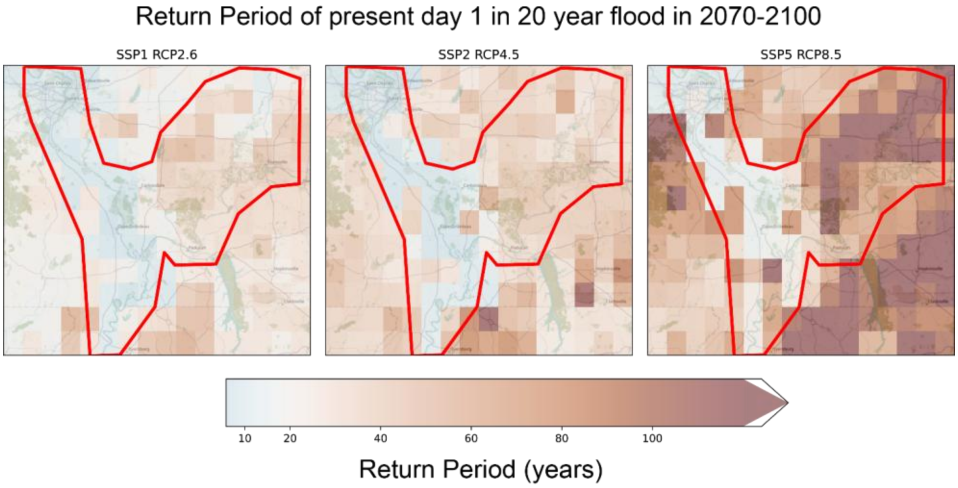

Extended Data Figure 2: *The return period of a present day 20-year flood event in 2071-2100 using 3 climate scenarios. Data based on flood model projections forced using the median of CMIP6 simulations as given in Hirabayashi et al (2021; ref. 7) (see Methods). The study area is bounded by the red lined polygon.*

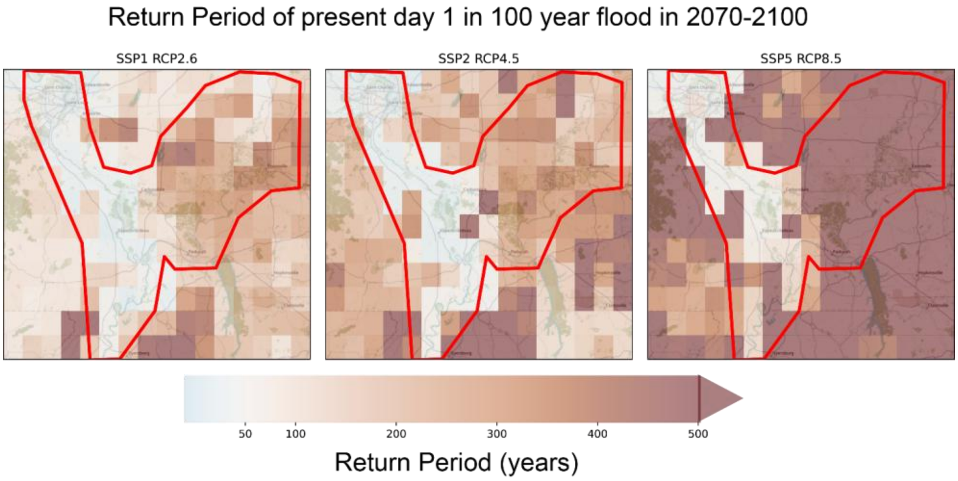

Extended Data Figure 3: *The return period of a present day 100-year flood event in 2071-2100 using 3 climate scenarios. Data based on flood model projections forced using the median of CMIP6 simulations as given in Hirabayashi et al (2021; ref. 7) (see Methods). The study area is bounded by the red lined polygon.*

Extended Data Table 1: *Annual Exceedance Probabilities (AEP) for the 2011 flood event as calculated by Driscoll et al (2014; ref. 39) at six gauging stations (Station ID locations are shown in Figure 1 of the main paper) located within the model domain. To estimate an overall AEP for the 2011 flood event, we take the mean values of the AEP for each of these six gauges. We therefore find an AEP of 1.2 with a 95% confidence interval of 0.75 and 3.85 for the 2011 flood. These AEP values correspond to ~1 in 80-year return period flood, with a 95% confidence interval ranging from ~1 in 25-year to ~1 in 130-year return periods. Note that gauges 3378500 and 3381700 have very large flows, with the AEP appearing to be ~0.1 as indicated by the growth curves from the stations.*

Gauge ID	Gauge Name	AEP	AEP 95%	AEP 5%
3377500	Wabash River at Mount Carmel, Illinois	2.1	0.4	4
3378500	Wabash River at New Harmony, Indiana	0.1	0.1	0.1
3381500	Little Wabash River at Carmi, Illinois	0.7	0.3	2.5
3381700	Ohio River at Old Shawneetown, Illinois	0.1	0.1	0.1
3399800	Ohio River at Smithland Dam, Smithland, Kentucky	0.2	1	9.5
3611500	Ohio River at Metropolis, Illinois	4	2.6	6.9
**Mean Values**		**1.2**	**0.75**	**3.85**

Extended Data Table 2: *Model skill scores for a range of commonly employed metrics (see Methods) as obtained for the simulated 1 in 75-year flood across the entire model domain and for a range of simulated bankfull return period values.*

Simulation Bankfull Return Period (BFRP)	Critical Success Index (CSI)	Hit Rate (HR)	Miss Rate (MR)	False Alarm Ratio (FAR)
1.1	0.584	0.988	0.012	0.412
1.5	0.59	0.967	0.033	0.398
2	0.595	0.96	0.038	0.378
5	0.602	0.902	0.098	0.356
10	0.592	0.855	0.145	0.342

Extended Data Table 3. *Simulated inundated areas and estimated people exposed for the model scenarios illustrated in Figure 4. These scenarios comprise: (1) a baseline of present-day climate and a bankfull channel conveyance capacity represented by the 2-year bankfull return period (BFRP) flow; (2) present-day climate with a BFRP at the upper bound (1.1 yrs) of present-day values estimated from the gauges in the study area; (3) present-day climate with a BFRP at the lower bound (0.3 years) of present-day values estimated from the gauges in the study area; (4) present-day climate with a BFRP set at the upper bound (2.1 years) of historical values estimated from gauges in the study area; (5) present-day climate with a BFRP set at the lower bound (1.2 years) of historical values estimated from gauges in the study area; (6) Future (2071-2100) climate change scenario (SSP 1 RCP 2.6) with a 2-year BFRP flow; (7) Future (2071-2100) climate change scenario (SSP 2 RCP 4.5) with a 2-year BFRP flow, and; (8) Future (2071-2100) climate change scenario (SSP5 RCP8.5) with a 2-year BFRP flow. Note that for the future climate scenarios, the present-day return periods of the two forcing flows are adjusted using change factors (Extended Data Figures 2 and 3).*

**Climate-Change and Channel Conveyance Scenarios**	**Forcing Flow Scenario**					
	**Relatively Frequent Flood (5-year RP)**		**Moderate Flood (20-year RP)**		**Large Flood (100-year RP)**	
	**Simulated Flood Extent (km²)**	**Change in Simulated Flood Extent (%)**	**Simulated Flood Extent (km²)**	**Change in Simulated Flood Extent (%)**	**Simulated Flood Extent (km²)**	**Change in Simulated Flood Extent (%)**
1. Present-day climate and 2-yr BFRP (baseline)	6,220		10,204	-	13,781	-
2. Present-day climate and 1.1-yr (upper observed) BFRP	9,306	49.6	12,289	20.4	15,058	9.3
3. Present-day climate and (lower observed) 0.3-yr BFRP	15,654	151.6	14,506	42.2	16,~~176~~697	~~58.5~~21.2 ~~17,308~~ ~~25.6~~
4. Present-day climate and 2.1-yr (upper historical) BFRP	5,509	-11.6	10,~~095~~290	-0.1~~.0~~	13,~~771~~752	-0.~~1~~3
5. Present-day climate and 1.2-yr (lower historical) BFRP	~~11,904~~8,864	~~16.6~~42.5	12,020	17.8	14,~~788~~884	~~7.3~~8.0
6. Future (2071-2100) climate (SSP1	-	-	9,288	-9.0	13,702	-0.6

Inserted Cells
Inserted Cells
Inserted Cells
Inserted Cells
Inserted Cells
Deleted Cells
Deleted Cells
Inserted Cells
Inserted Cells
Inserted Cells
Inserted Cells
Inserted Cells
Inserted Cells

RCP2.6) and 2-year BFRP						
7. Future (2071-2100) climate (SSP2 RCP4.5) and 2-year BFRP	-	-	9,864	-3.3	13,964	1.3
8. Future (2071-2100) climate (SSP5 RCP8.5) and 2-year BFRP	-	-	12,630	23.8	15,177	10.1
			Population Exposed	Change in Population Exposed (%)	Population Exposed	Change in Population Exposed (%)
1. Present-day climate and 2-yr BFRP (baseline)	41,956		119,059	-	205,817	-
2. Present-day climate and 1.1-yr (upper observed) BFRP	104,588	149.2	181,283	52.3	237,162	15.2
3. Present-day climate and (lower observed) 0.3-yr BFRP	~~259,613~~197,865	~~118.1~~471.6	~~316,856~~ 278,244	~~54.0~~133.7	273,296	32.8
4. Present-day climate and 2.1-yr (upper historical) BFRP	~~117,175~~49,272	~~-1.6~~17.4	~~202,749~~118,571	-~~1.5~~0.4	206,630	-0.4
5. Present-day climate and 1.2-yr (lower historical) BFRP	~~170,542~~93,832	~~43.2~~223.6	~~230,528~~172,358	~~12.0~~44.8	232,732	13.1
6. Future (2071-2100) climate (SSP1 RCP2.6) and 2-year BFRP	-	-	60,615	-49.1	175,595	-14.7
7. Future (2071-2100) climate	-	-	63,204	-46.9	177,770	-13.6

Inserted Cells

Inserted Cells

Inserted Cells

Inserted Cells

Inserted Cells

Inserted Cells

(SSP2 RCP4.5) and 2-year BFRP						
8. Future (2071-2100) climate (SSP5 RCP8.5) and 2-year BFRP	-	-	115,177	-3.3	245,801	19.4

Extended Data Table 4 *Calculations of time-evolution of flood hazard (inundation extent) and ~~risk~~impact (populations exposed) for the relatively frequent (5-year) driving flood discharge and as forced by observed historical changes in channel conveyance capacity versus future climate changes. The numbered scenarios are defined in the caption to Extended Data Table 3.*

Scenario	Flood Extent Calculations			Population Exposure Calculations		
	Simulated Flood Extent (km^2)	Change in Simulated Flood Extent (km^2)	Rate of Change of Simulated Flood Extent (km^2/decade)	People Exposed	Change in People Exposed	Rate of Change of Exposure (people/decade)
1. Present-day climate and 2-yr BFRP (baseline)	6,220	n/a	n/a	41,956	n/a	n/a
4. Start point of channel change scenario using present-day climate and 2.1-year (historical) BFRP	5,509	n/a	n/a	49,272	n/a	n/a
5. End point of channel change scenario using present day climate and 1.2-year (historical) BFRP	8,864	+3,355[1]	**+781[3]**	93,832	+44,560[1]	**+10,374[3]**

Notes on calculations:

1. computed using difference between scenario 5 and scenario 4.

Extended Data Table 5 *Calculations of time-evolution of flood hazard (inundation extent) and impact (populations exposed) for the moderate (20-year) driving flood discharge and as forced by observed historical changes in channel conveyance capacity versus future climate changes. The numbered scenarios are defined in the caption to Extended Data Table 3*.

**Scenario**	**Flood Extent Calculations**			**Population Exposure Calculations**		
	**Simulated Flood Extent (km²)**	**Change in Simulated Flood Extent (km²)**	**Rate of Change of Simulated Flood Extent (km²/decade)**	**People Exposed**	**Change in People Exposed**	**Rate of Change of Exposure (people/decade)**
1. Present-day climate and 2-yr BFRP (baseline)	10,204	n/a	n/a	119,059	n/a	n/a
4. Start point of channel change scenario using present-day climate and 2.1-year (historical) BFRP	10,~~095~~290	n/a	n/a	~~117,175~~118,571	n/a	n/a
5. End point of channel change scenario using present day climate and 1.2-year (historical) BFRP	~~11,904~~12,020	+1,~~809[1]~~730[1]	**+~~421[3]~~403[3]**	~~170,542~~172,358	+53,~~367[1]~~787[1]	**+12,~~411[3]~~523[3]**
6. Future (2071-2100) climate (SSP1 RCP2.6) and 2-year BFRP	9,288	-916[2]	**-150[4]**	60,615	-58,444[2]	**-9,741[4]**
7. Future (2071-2100) climate (SSP2 RCP4.5) and 2-year BFRP	9,864	-340[2]	**-56[4]**	63,204	-55,855[2]	**-9,309[4]**
8. Future (2071-2100) climate (SSP5 RCP8.5) and 2-year BFRP	12,630	+2,426[2]	**+404[4]**	115,177	-3,882[2]	**-647[4]**

Notes on calculations:

1. computed using difference between scenario 5 and scenario 4.
2. computed using difference between this scenario and the baseline scenario 1.
3. computed by dividing the estimated change (reported in cell to left) by the 43-year period of gauge record, and then converting the annual rate so-obtained to a decadal rate.
4. computed by dividing the estimate change (reported in cell to left) by the 60-year period between the present-day (2025) and the mid-point (2085) of the future climate, and then converting the annual rate so obtained to a decadal rate.

Extended Data Table ~~5~~6 *Calculations of time-evolution of flood hazard (inundation extent) and ~~risk~~impact (populations exposed) for the ~~moderate~~large (100-year) driving flood discharge and as forced by observed historical changes in channel conveyance capacity versus future climate changes. The numbered scenarios are defined in the caption to Extended Data Table 3.*

Scenario	Flood Extent Calculations			Population Exposure Calculations		
	Simulated Flood Extent (km^2)	Change in Simulated Flood Extent (km^2)	Rate of Change of Simulated Flood Extent (km^2/decade)	People Exposed	Change in People Exposed	Rate of Change of Exposure (people/decade)
1. Present-day climate and 2-yr BFRP (baseline)	13,781	n/a	n/a	205,817	n/a	n/a
4. Start point of channel change scenario using present-day climate and 2.1-year (historical) BFRP	13,~~771~~752	n/a	n/a	~~202,749~~206,630	n/a	n/a
5. End point of channel change scenario using present day climate and 1.2-year (historical) BFRP	14,~~788~~884	+1,~~017~~[1]127[1]	**+~~237~~[3]262[3]**	~~230,528~~232,732	+~~24,711~~[1]26,102[1]	**+~~5,747~~[3]6,077[3]**
6. Future (2071-2100) climate (SSP1 RCP2.6) and 2-year BFRP	13,702	-79[2]	**-13[4]**	175,595	-30,222[2]	**-5,037[4]**
7. Future (2071-2100) climate (SSP2 RCP4.5) and 2-year BFRP	13,964	+183[2]	**+31[4]**	177,770	-28,047[2]	**-4,675[4]**
8. Future (2071-2100) climate (SSP5 RCP8.5) and 2-year BFRP	15,177	+1,396[2]	**+233[4]**	245,801	+39,984[2]	**+6,664[4]**

Notes on calculations:

1. computed using difference between scenario 5 and scenario 4.
2. computed using difference between this scenario and the baseline scenario 1.
3. computed by dividing the estimated change (reported in cell to left) by the 43-year period of gauge record, and then converting the annual rate so-obtained to a decadal rate.
4. computed by dividing the estimate change (reported in cell to left) by the 60-year period between the present-day (2025) and the mid-point (2085) of the future climate, and then converting the annual rate so obtained to a decadal rate.
